# Novelties in *Selaginella* (Selaginellaceae – Lycopodiophyta), with emphasis on Brazilian species

**DOI:** 10.3897/phytokeys.57.6489

**Published:** 2015-12-15

**Authors:** Iván A. Valdespino

**Affiliations:** 1 Departamento de Botánica, Facultad de Ciencias Naturales, Exactas y Tecnología, Universidad de Panamá, Apartado Postal 0824-00073, Panama

**Keywords:** Deforestation, Endangered, lycophytes, Morro de Cubiçado, Serra do Mar

## Abstract

In this paper, I describe five new species of *Selaginella* from Brazil (*Selaginella nanuzae*, *Selaginella neospringiana*, *Selaginella pellucidopunctata*, *Selaginella stomatoloma*, and *Selaginella trygonoides*), compare them to morphologically similar species, and provide a preliminary conservation status assessment for each. The new species are illustrated with scanning electron photomicrographs of stem sections, leaves, and spores, when available. Also discussed in this paper are ten species, mainly from Brazil and with new distribution records, and the forthcoming resurrection of three species also occurring in Brazil. Three further non-native and presumed naturalized species are recognized in Brazil, and publication of one additional taxon is planned. Eighty-six *Selaginella* species are now known from Brazil and, of these, 80 are native (including 26 / 32.5%, endemic), and six are introduced. Brazil and Mexico have the second highest number of native *Selaginella* species in the Neotropics after Venezuela, which is estimated to have about 100. Of the newly documented species, *Selaginella cabrerensis* is now known to occur in French Guiana, Brazil, and Bolivia, in addition to Colombia, and *Selaginella arroyoana* and *Selaginella chiquitana* are synonymized under it. Likewise, *Selaginella potaroensis* is also recorded from Costa Rica and Brazil, and *Selaginella seemannii* from Panama and Brazil. Finally, leaf marginal stomata are reported on the newly described species and their functionality is discussed under *Selaginella stomatoloma*.

## Abstract (es)

En este artículo describo cinco nuevas especies de *Selaginella* de Brasil (*Selaginella nanuzae*, *Selaginella neospringiana*, *Selaginella pellucidopunctata*, *Selaginella stomatoloma* y *Selaginella trygonoides*), las cuales comparo con especies morfológicamente similares y estimo, preliminarmente, su estado de conservación. Las nuevas especies las ilustro con fotomicrografías al microscopio electrónico de barrido de secciones de los tallos, hojas y esporas (cuando están disponibles). Diez especies adicionales son discutidas en este artículo con nuevos registros, mayormente para Brasil, y la resurrección de otras tres también presentes en este país es anunciada. Tres especies introducidas y presuntamente naturalizadas adicionales son documentadas en Brasil, mientras que se anuncia la publicación próximamente de una especie nueva que también ocurre en este país. Por lo tanto, la ocurrencia de 86 especies de *Selaginella* en Brasil es conocida ahora, de las cuales 80 son nativas (26 de ellas o el 32.5% son endémicas) y seis son introducidas. Brasil y México tienen el segundo mayor número de especies nativas de *Selaginella* en el Neotrópico, después de Venezuela que se estima que posee unas 100. De las especies documentadas por primera vez, *Selaginella cabrerensis* se reconoce que está distribuida en Guayana Francesa, Brasil y Bolivia, además de Colombia, y se consideran formalmente como sinónimo de ésta a *Selaginella arroyoana* y a *Selaginella chiquitana*. Adicionalmente, *Selaginella potaroensis* es documentada en Costa Rica y Brasil, al igual que lo es *Selaginella seemannii* en Panamá y Brasil. Finalmente, la presencia de estomas en los márgenes de las hojas se reporta en las cinco nuevas especies descritas y su funcionalidad se discute bajo *Selaginella stomatoloma*.

## Introduction

In a recent paper, Valdespino et al. (2015) described seven new species of *Selaginella* from Brazil, raising the number of the known native species of that country to 58, and suggested that additional ones could be uncovered as further work in this genus continued. That prediction has proven accurate: Valdespino (2015a) has proposed *Selaginella boomii* Valdespino, a new species widely distributed in South America, including Brazil. In addition, Goés-Neto et al. (2015) described *Selaginella salinoi* Goés-Neto & G. Heringer, and Valdespino (2015b) published, *Selaginella monticola* Valdespino. These two species are restricted to Brazil. As part of my revisionary work on Brazilian *Selaginella* initiated in 1992, studying collections in R and RB, I now describe five additional new heteromorphic species from Brazil: *Selaginella nanuzae* Valdespino, *Selaginella neospringiana* Valdespino, *Selaginella pellucidopunctata* Valdespino, *Selaginella stomatoloma* Valdespino, and *Selaginella trygonoides* Valdespino. Moreover, 10 species are now recorded or confirmed for the first time mainly for that country: *Selaginella beitelii* A.R. Sm., *Selaginella cabrerensis* Hieron., *Selaginella falcata* (P. Beauv.) Spring, *Selaginella lechleri* Hieron., *Selaginella microdonta* A.C. Sm., *Selaginella potaroensis* Jenman, *Selaginella seemannii* Baker, *Selaginella umbrosa* Lem. ex Hieron., *Selaginella vernicosa* Baker, and *Selaginella wurdackii* Alston. Another species previously known to occur in Venezuela and just recently recognized in Brazil, included in Table 1 as *Selaginella* sp. A, will be described in the near future (Valdespino in prep.). Three species previously recorded from Brazil (i.e., *Selaginella chromatophylla* Silveira, *Selaginella deltoides* A. Braun [see also Valdespino in press], and *Selaginella glazioviana* Hieron. [see discussion under *Selaginella trygonoides*]), which were placed in synonymy by other authors (e.g., Alston et al. 1981), are in the midst of being reinstated in a separate paper (Valdespino, in prep.). Three non-native species are vouchered for the first time in Brazil: *Selaginella braunii* Baker, *Selaginella pallescens* (C. Presl) Spring, and *Selaginella willdenowii* (Desv.) Baker. Of the newly recorded species, *Selaginella cabrerensis* (with updated synonymy), *Selaginella potaroensis*, and *Selaginella seemannii* are also documented for other neotropical countries.

The novelties reported here increase the number of *Selaginella* species in Brazil to 86 (see Table 1), of which 80 are native and six are introduced and presumed naturalized. Among the native species, 26 are endemic (see taxa with an asterisk in Table 1). These statistics represent an increase of 44% and 35% in the number of *Selaginella* species recorded in Brazil over previous accounts by Alston et al. (1981) and Hirai (2015), respectively, and also increases the percentage of estimated endemic species from 27.2% (Prado and Hirai 2014) to 32.5%. After Venezuela, which is estimated to have ca. 100 species (Valdespino in press), Brazil (Table 1) and Mexico also with 80 native species (Mickel et al. 2004) have the second highest diversity of *Selaginella* in the Neotropics. With these new data, and based on information by Prado and Hirai (2014) for Brazilian lycophytes and ferns diversity, *Selaginella* is now the third most diverse genus and twelfth in endemism for that country.

Following Jermy’s (1986, 1990) subgeneric classification, the newly described *Selaginella nanuzae*, *Selaginella neospringiana*, *Selaginella stomatoloma*, and *Selaginella trygonoides* belong to subg. *Stachygynandrum* because their strobili are quadrangular and comprise monomorphic sporophylls, while *Selaginella pellucidopunctata*, with dimorphic sporophylls, has dorsiventral strobili and is a member of subg. *Heterostachys*.

**Table 1.** Checklist of *Selaginella* in Brazil [species included are documented by a single selected voucher for the country or by a published reference; Asterisk denotes endemic species].

N˚	*Selaginella* species	Voucher examined	Observation
1	*Selaginella alstonii* G. Heringer, Salino & Valdespino*	Minas Gerais: *Almeida et al. 533* (BHCB, PMA)	
2	*Selaginella amazonica* Spring	Amazonas: *Luetzelburg 23646* (R)	
3	*Selaginella anceps* (C. Presl) C. Presl	Acre: *Daly et al. 8139* (NY, MO)	
4	*Selaginella applanata* A. Braun	Amazonas: *Prance et al. 14339* (F)	
5	*Selaginella articulata* (Kunze) Spring	—	Hirai (2015)
6	*Selaginella asperula* Spring	Acre: *Daly 7573* (NY)	
7	*Selaginella bahiensis* Spring*	Bahia: *Thomas et al. 14086* (MO, NY)	
8	*Selaginella blepharodella* Valdespino*	Bahia: *Moraes & van der Werff 2933* (MO, PMA, UC)	
9	*Selaginella beitelii* A.R. Sm.	Amazonas: *Carvalho et al. 353* (PMA)	Newly reported
10	*Selaginella boomii* Valdespino	Pará: *Plowman et al. 8563* (F, GH, MG, NY, US)	
11	*Selaginella brevifolia* Baker	Amazonas: *Spruce 2547* (BM, BR, GH, E, G, K, OXF, P, RB, US)	
12	*Selaginella breynii* Spring	Amapá: *Egler & Irwing 46420* (NY)	
13	*Selaginella cabrerensis* Hieron.	Goiás: *Irwin et al. 15552* (NY)	Newly confirmed
14	*Selaginella calceolata* Jermy & J.M. Rankin	Amazonas: *Spruce 2861* (G, GH, P, RB, W)	
15	*Selaginella crinita* Valdespino*	Bahia: *Harley & Taylor 27048* (NY, PMA)	
16	*Selaginella chromatophylla* Silveira*	Bahia: *Moraes & van der Werff 2861* (MO, PMA, UC)	To be resurrected
17	*Selaginella coarctata* Spring	Amazonas: *Rosa & Lia 2339* (NY)	
18	*Selaginella conduplicata* Spring	Amazonas: *Todzia et al. 2262* (NY)	
19	*Selaginella contigua* Baker*	Rio de Janeiro: *Sylvestre et al. 1874* (NY)	
20	*Selaginella convoluta* (Arn.) Spring	Rio de Janeiro: *Braga 7652* (NY)	
21	*Selaginella decomposita* Spring*	Bahia: *Thomas et al. 14223* (NY)	
22	*Selaginella deltoides* A. Braun	Amazonas: *Luetzelburg 23710* (M, R)	To be resurrected
23	*Selaginella dendricola* Jenman	Amazonas: *Spruce 2535* (OXF, P)	
24	*Selaginella epirrhizos* Spring	Amazonas: *Cid et al. 605* (NY)	
25	*Selaginella erectifolia* Spring*	Rio de Janeiro: *Glaziou 2242* (B, BR, K, NY, P)	
26	*Selaginella erythropus* (Mart.) Spring	Mato Grosso: *Windisch et al. 6758* (NY)	
27	*Selaginella exaltata* (Kunze) Spring	Amazonas: *Prance et al. 7626* (NY)	
28	*Selaginella falcata* (P. Beauv.) Spring	Amapá: *Bastos 2070* (RB)	Newly reported
29	*Selaginella flagellata* Spring	Pará: *Sperling et al*, 5589 (NY)	
30	*Selaginella flexuosa* Spring	Bahia: *Edwards 2431* (NY)	
31	*Selaginella fragilis* A. Braun	Amazonas: *Ferreira et al. 7930* (NY)	
32	*Selaginella glazioviana* Hieron.*	Rio de Janeiro: Glaziou 7280 (B, BM)	To be resurrected
33	*Selaginella gynostachya* Valdespino	Pará: *Maciel & Pietrobom 1032* (MG, PMA)	
34	*Selaginella haematodes* (Kunze) Spring	Rondônia: *Teixeira et al. 427* (NY)	
35	*Selaginella homaliae* A. Braun	Amazonas: *Stevenson & Ramos 978b* (NY)	
36	*Selaginella jungermannioides* (Gaudich.) Spring*	Rio de Janeiro: *Rose & Russell 20349* (NY)	
37	*Selaginella kochii* Hieron.	Amazonas: *Alencar 327* (NY)	
38	*Selaginella lechleri* Hieron.	Acre: *Jangoux et al. 85-104* (NY)	Newly reported
39	*Selaginella macrostachya* (Spring) Spring*	São Paulo: *Handro 2059* (NY)	
40	*Selaginella marginata* (Humb. & Bonpl. ex Willd.) Spring	Brasilia, DF: *da Silva et al. 3532* (NY)	
41	*Selaginella mendocae* Hieron.*	Rio de Janeiro: *Brade 11664* (R)	
42	*Selaginella microdonta* A.C. Sm.	Amazonas: *Cavalcante 3056* (MG)	Newly reported
43	*Selaginella microphylla* (Kunth) Spring	Rio Grande do Sul: *Vital & Buck 12194* (NY)	
44	*Selaginella minima* Spring	Goiás: Anderso*n 7863* (K, NY)	
45	*Selaginella monticola* Valdespino*	São Paulo: *Salino 2980* (PMA)	
46	*Selaginella mucronata* G. Heringer, Salino & Valdespino*	Espírito Santo: *Salino et al. 13686* (BHCB, PMA)	
47	*Selaginella mucugensis* Valdespino*	Bahia: *Giulietti et al. [CFCR 1430*] (NY, PMA)	
48	*Selaginella muscosa* Spring	Rio de Janeiro: *Brade 17189* (BM, G, MG, MO, RB)	
49	*Selaginella nanuzae* Valdespino*	São Paulo: *Salino et al. 7788* (PMA)	Newly described
50	*Selaginella neospringiana* Valdespino*	Rio de Janeiro: *Glaziou 11723* (BM, C, P, PM, US)	Newly described
51	*Selaginella palmiformis* Alston ex Crabbe & Jermy	Amazonas: *Campbell et al. P21811* (GH, K, MO, NY, R, S)	
52	*Selaginella parkeri* (Hook. & Grev.) Spring	Acre: *Silveira et al. 1273* (NY)	
53	*Selaginella pellucidopunctata* Valdespino*	Alagoas: *Oliveira 1094* (PMA, UFP)	Newly described
54	*Selaginella porelloides* (Lam.) Spring	Mato Grosso: Anderson 9901 (AAU, F, NY, UC)	
55	*Selaginella potaroensis* Jenman	Roraima: *Prance et al. 9995* (NY)	Newly reported
56	*Selaginella producta* Baker	Bahia: *Thomas et al. 10610* (NY)	
57	*Selaginella radiata* (Aubl.) Spring	Pará: *Sperling et al. 5715* (NY)	
58	*Selaginella revoluta* Baker	Amazonas: *Maguire et al. 60296* (MO, NY)	
59	*Selaginella salinoi* Goés-Neto & G. Heringer*	Espírito Santo: *Souza et al. 1462* (PMA)	
60	*Selaginella saltuicola* Valdespino*	Mato Grosso: *Prance et al. 19126* (INPA, NY, PMA)	
61	*Selaginella sandwithii* Alston	Amapá: *Irwin et al. 47416* (U, US).	
62	*Selaginella seemannii* Baker	Roraima: *Edwards & Millikin 2541* (NY)	Newly reported
63	*Selaginella sellowii* Hieron.	Rio Grande do Sul: *Leite 2381* (NY)	
64	*Selaginella sematophylla* Valdespino, G. Heringer & Salino*	Minas Gerais: *Brade & Barbosa 17953* (BM, MO, NY, PMA, RB)	
65	*Selaginella simplex* Baker	Goiás: *Anderson 8187* (NY)	
66	*Selaginella stomatoloma* Valdespino*	Pará: *Almeida et al. 2518* (PMA)	Newly described
67	*Selaginella suavis* (Spring) Spring	Espiritu Santo: *Mexia 4072* (MO)	
68	*Selaginella sulcata* (Desv.) Spring ex Mart.	Rio de Janeiro: *Araujo & Carauta 1452* (NY)	
69	*Selaginella tenella* (P. Beauv.) Spring	Mato Grosso: Lindman A3495 (B, S)	
70	*Selaginella tenuissima* Fée*	Minas Gerais: *Vital & Buck 11544* (NY)	
71	*Selaginella terezoana* Bautista	Roraima: *Terezo 32* (IAN)	
72	*Selaginella trisulcata* Aspl.	Pará: *Prance & Pennington* (BM)	Alston et al. (1981: 320)
73	*Selaginella trygonoides* Valdespino*	Minas Gerais: *Almeida et al. 1994* (PMA)	Newly described
74	*Selaginella tuberculata* Spruce ex Baker	Amazonas: *Stevenson et al. 978a* (NY)	
75	*Selaginella umbrosa* Lem. ex Hieron.	Paraná: *Sucre et al.* 9778 (RB)	Newly reported
76	*Selaginella valida* Alston*	Paraná: *Matos & Schwartsburd 826* (NY)	
77	*Selaginella vernicosa* Baker	Roraima: *Luetzelburg 21631* (R)	Newly reported
78	*Selaginella vestiens* Baker*	Minas Gerais: *Mexia 5832* (CAS, GH, MICH, MO, S, U)	
79	*Selaginella wurdackii* Alston	Roraima: *Carvalho et al. 233* (INPA, PMA)	Newly reported
80	*Selaginella* sp. A.	Roraima: *Carvalho et al. 374* (INPA, PMA)	To be described by Valdespino with epithet “*psittacorrhincha*”
**Introduced species**			
81	*Selaginella braunii* Baker	Rio de Janeiro: *Winter 71* (NY, RB)	Newly reported (escape from cultivation at RB?)
82	*Selaginella kraussiana* A. Braun	São Paulo: *Hoehne 222* (NY)	
83	*Selaginella pallescens* (C. Presl) Spring	Santa Catharina: *Schmalz 148* (NY)	Newly reported (escape from cultivation?)
84	*Selaginella plana* (Desv.) Hieron.	São Paulo: *Wells-Windisch*, 583 (HB-n.v.)	Hirai (2015)
85	*Selaginella vogelii* Spring	Rio de Janeiro: *Engelmann 116* (NY)	
86	*Selaginella willdenowii* (Desv.) Baker	Pará: *Killip & A.C. Sm.th 30345* (NY)	Newly reported

## Material and methods

This study is based on examination of herbarium specimens from AAU, B, BHCB, BM, BR, C, CAS, COL, CR, E, F, G, GH, IAN, INPA, K, L, M, MG, MICH, MO, NY, OXF, P, PMA, RB, R, S, U, UC, UFP, US, W, and digitized images from B, BM, C, P, and UC (herbarium acronyms follow Thiers 2015). Further digitized *Selaginella* specimens from Brazil were consulted from RB (JBRJ 2015), Reflora (2015), and Species Link (2015) virtual herbaria. Additionally, stem sections and spore samples from selected specimens were viewed with Scanning Electron Microscope (SEM) to determine upper and lower surfaces of leaves and spore sculpturing patterns and diameter (when available). The SEM study and measurement of leaves was conducted following Valdespino et al. (2014, 2015). Spore sculpturing and leaf terminology follow Valdespino et al. (2015 and references therein). The raw SEM images were processed with Adobe Photoshop to make the background black, adjust brightness and contrast, and for assembly in multipart figures according to species. Figures are provided only for new species, although additional SEM digitized images were taken for comparison from some other species discussed.

Species descriptions are given only for new taxa and follow the order of characters and states used by Valdespino et al. (2015 and references therein). Entries for taxa newly recorded for Brazil and other countries are provided only for native species in a concise form, as most will be dealt with in separate papers, while non-native and yet unpublished ones are documented within Table 1 with herbarium vouchers examined or published references. Unique identifier numbers (usually barcode numbers) in square brackets were provided for type specimens when available.

Conservation statuses were assessed for only new taxa and follow the IUCN Red list Categories and Criteria version 3.1, second edition (IUCN 2012).

## Taxonomy

### Selaginella nanuzae

Taxon classificationPlantaeSelaginellalesSelaginellaceae

Valdespino sp. nov.

urn:lsid:ipni.org:names:77151571-1

Figures 1 , 2 , 3

#### Diagnosis.

*Selaginella nanuzae* differs from typical *Selaginella contigua* Baker by its coriaceous (vs. chartaceous) leaves with the upper surfaces shiny and bumpy (vs. dull and smooth to slightly corrugate), the acroscopic margins of the lateral leaves and both margins of the median and axillary leaves broadly (vs. faintly) hyaline, median leaves with long-aristate (vs. acute to short-acuminate) apices, each arista 0.4–0.6 mm (vs. acumen 0.05–0.2 mm), midribs straight (vs. arcuate) with the outer bases auriculate (vs. lacking auricles) and bearing10–18 long-cilia (vs. outer bases ciliate with 2–5 cilia), and the lateral leaves 2.5–3.0 × 1.0–1.5 mm (vs. 7.0–10 × 2.0–2.7 mm).

#### Type.

**BRAZIL**. São Paulo: [Mpio.] Ubatuba, Parque Estadual da Serra do Mar, Núcleo Picinguaba, trail to Pico Corcovado, 23°26'56.6"S, 45°11'35.8"W, 450 m, 1 Nov 2001, *A. Salino, V.A.O. Dittrich, P.O. Morais, F.A. Carvalho, L.C.R.S. Teixeira & A.M. Oliveira 7788* (holotype: PMA! [PMA103268]; isotype: BHCB [BHCB65041]-n.v.).

#### Description.

*Plants* terrestrial. *Stems* decumbent to ascending or suberect, stramineous, 3–5 cm long, 0.5–1.0 mm diam., non-articulate, not flagelliform or stoloniferous, 2–3-branched. *Rhizophores* ventral or ventro-axillary, borne on proximal ½ of stems, stout, 0.5–1.0 mm diam. *Leaves* heteromorphic throughout, coriaceous, strongly imbricate, upper surfaces bumpy and green, lower surfaces corrugate to striate and silvery green. *Lateral leaves* ascending, ovate-oblong or ovate, 1.5–3.0 × 0.9–1.5 mm; bases rounded to semicordate, acroscopic bases strongly overlapping stems, basiscopic bases free from stems; acroscopic margins broadly hyaline in a band 5–10 cells wide with the cells elongate, straight-walled and papillate parallel to margins, papillae in 1 row over each cell lumen, long-ciliate along proximal ¾, otherwise denticulate distally, basiscopic margins greenish, comprising quadrangular, sinuate-walled, glabrous and papillate cells, long-ciliate along proximal ⅛, otherwise entire distally or denticulate on distal ⅛; apices falcate and acute or gradually tapering and acute, variously tipped by 1–3 teeth; upper surfaces comprising quadrangular or rounded, sinuate-walled cells (often difficult to distinguish because of waxy deposits), many of these covered by 6–40 papillae, without idioblasts or stomata, lower surfaces comprising elongate, sinuate-walled cells, with many of these papillate and idioblast-like on both side of the midribs (more so on acroscopic halves of the laminae), papillae in 1–3 rows (or rows not clearly distinguishable) over each cell lumen, with stomata in 2–5 rows along midribs and along distal ⅙ of basiscopic margins. *Median leaves* ascending, ovate to ovate-elliptic or orbicular, 1.2–1.8 × 0.6–1.2 mm; bases truncate or oblique with outer bases auriculate, auriculae tufted with 10–18 cilia; margins broadly hyaline in a band 2–8 cells wide, the cells elongate, straight-walled and papillate parallel to margins, papillae in 1 row over each cell lumen, long-ciliate throughout and more obviously so on outer margins (cilia on outer margins half to one time longer than the inner cilia); apices long-aristate, each arista ⅓–½ the length of the laminae (0.4–0.9 mm), variously tipped by 1–3 teeth; both surfaces without conspicuous idioblasts, upper surfaces comprising quadrangular, rectangular or rounded, sinuate-walled cells (often difficult to distinguish because of waxy deposits), many of these covered by 7–25 papillae, with stomata in 2–5 rows along distal ½ of the midribs, few on submarginal portion along proximal ⅕ of outer halves of the laminae, lower surfaces comprising elongate, sinuate-walled cells, without stomata. *Axillary leaves* ovate or ovate-lanceolate, 1.5–2.6 × 0.8–1.1 mm; bases rounded; margins broadly hyaline, long-ciliate along proximal ⅓, short-ciliate on central ⅓, otherwise dentate on distal ⅓; apices acute to broadly acute, variously tipped by 1–3 teeth; both surfaces as in lateral leaves. *Strobili* terminal on main stem and branch tips, loosely quadrangular, 0.4–1.6 cm. *Sporophylls* monomorphic, without a laminar flap, each with a strongly developed and seemingly glabrous keel along midribs, broadly ovate to ovate-deltate, 1.0–1.2 × 0.6–1.1 mm; bases truncate; margins broadly hyaline, dentate to short-ciliate; apices short-acuminate to cuspidate, each acumen (cusp) 0.1–0.2 mm, tipped by 1–3 teeth; *dorsal sporophylls* with upper surfaces green and cells as in median leaves, lower surfaces silvery green and comprising elongate, sinuate-walled cells; *ventral sporophylls* with both surfaces hyaline or greenish hyaline, comprising elongate, sinuate-walled cells. *Megasporangia* in 2 ventral rows; *megaspores* yellow, rugulate-reticulate on proximal faces with verrucate, perforate, and echinulate microstructure, reticulate on distal faces with verrucate and perforate microstructure (Fig. 3A–D), 260–355 µm. *Microsporangia* in 2 dorsal rows; *microspores* orange, rugulate on proximal faces with echinulate microstructure, capitate or clavate on distal faces with each caput and the rest of the surface with echinulate microstructure (Fig. 3E, F), 25–30 µm.

#### Habitat and distribution.

*Selaginella nanuzae* grows in dense premontane wet forests at 450 m in Atlantic forest vegetation. It is known only along the trail to Morro Corcovado in Parque Estadual da Serra do Mar, São Paulo.

#### Etymology.

This species is named for Professor, Dra. Nanuza Luiza de Menezes, an outstanding Brazilian botanist, who has been instrumental in advancing botanical and conservation sciences in her country and, in the course of her career, has mentored new generations of botanists at the University of São Paulo. Through her involvement with the Latin American Botanical Association, in 1992, I attended a plant morphology and anatomy course with emphases in taxonomy and evolution at that University; this led to my first exposure to, and study of, Brazilian *Selaginella*.

#### Conservation status.

*Selaginella nanuzae* was collected in a state park, where it may be adequately conserved. Nevertheless, given that there is large visitation along the trails of this park, particularly to ascend to Morro Corcovado where the only two known collections of the new taxa were made, and that some degree of deforestation occurs there, it is considered Vulnerable (VU).

#### Additional specimen examined

**(paratype). BRAZIL**. **São Paulo**: Mpio. Ubatuba, Morro Corcovado, 8 Sep 1998, *Ribas & Dittrich 2729* (NY).

#### Discussion.

**Figure 1.**
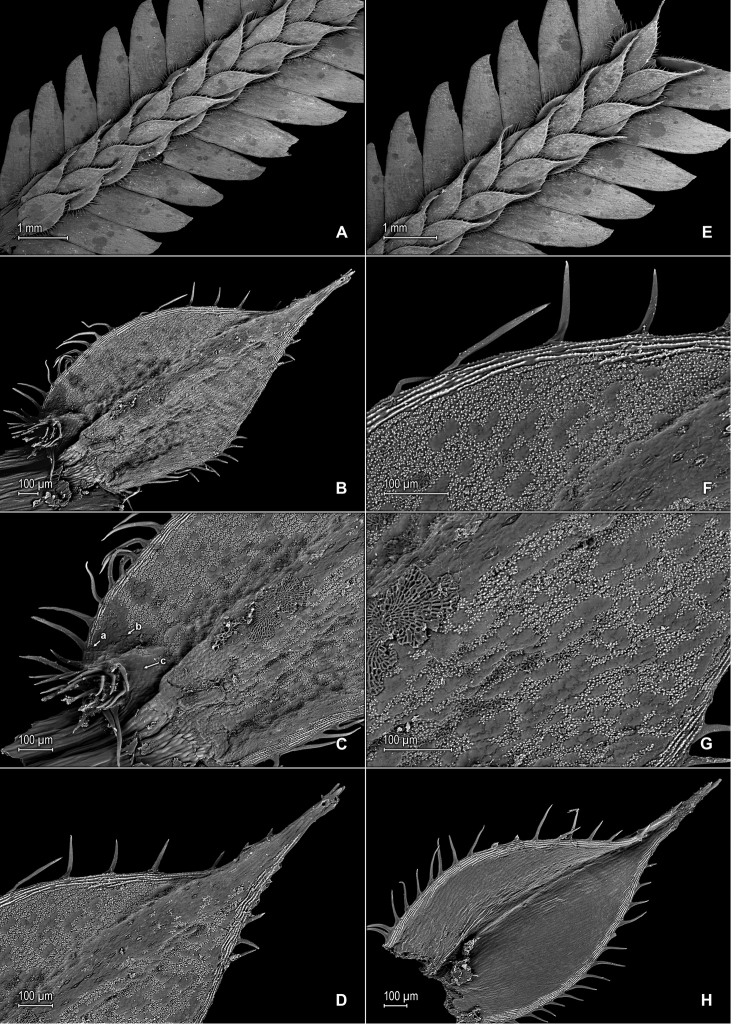
*Selaginella nanuzae* Valdespino. **A** Section of upper surface of stem **B** Upper surface of median leaf **C** Close-up of base and proximal portion of median leaf, upper surface; note marginal (a) and submarginal (b) stoma, and outer base tufted with long cilia (c) **D** Close-up of distal portion and apex of median leaf, upper surface **E** Section of upper surface of stem **F** Close-up of outer half of median leaf, upper surface **G** Close-up-of inner half of median leaf, upper surface **H** Lower surface of median leaf. **A–H** taken from the holotype, *Salino et al. 7788* (PMA).

**Figure 2.**
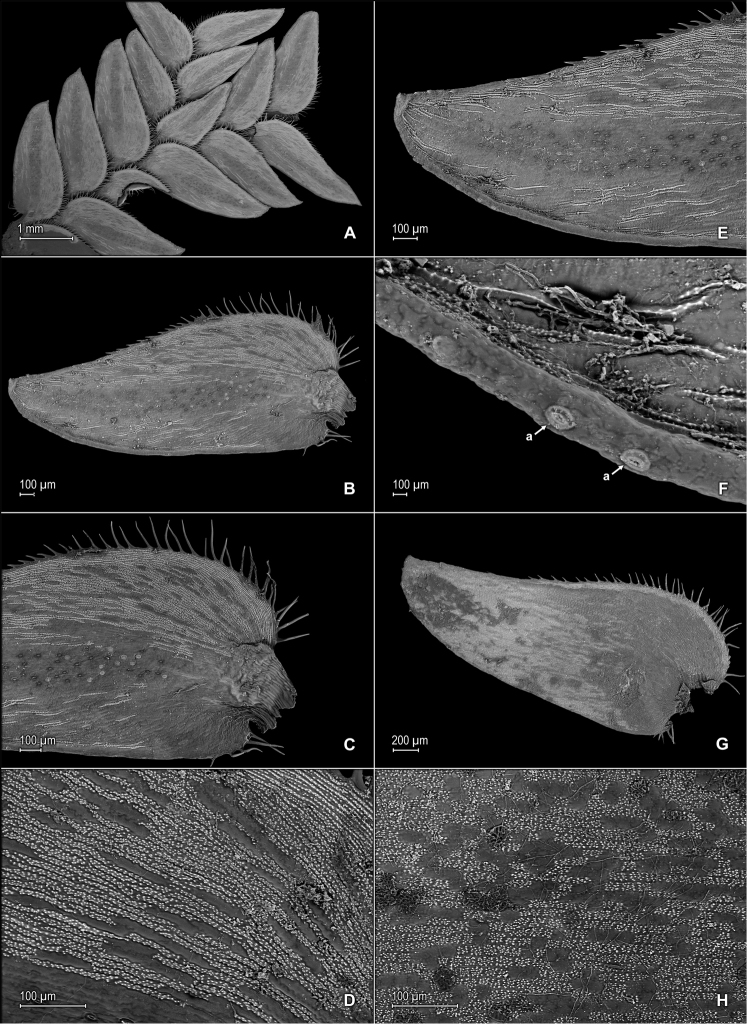
*Selaginella nanuzae* Valdespino. **A** Section of lower surface of stem **B** Lower surface of lateral leaf **C** Close-up of base and distal portion of lateral leaf, lower surface **D** Close-up of acroscopic half of lateral leaf near base, lower surface **E** Close-up of distal portion and apex of lateral leaf, lower surface **F** Close-up of portion of basiscopic margin of lateral leaf near apex, lower surface; note marginal stoma (a) **G** Upper surface of lateral leaf **H** Close-up of upper surface of lateral leaf. **A–H** taken from the holotype, *Salino et al. 7788* (PMA).

*Selaginella nanuzae* is characterized by the upper surfaces of the leaves shiny (due to waxy deposits) and bumpy (Figs 1, 2), the median leaves long-aristate (Fig. 1A, B, E, F), the acroscopic margins of the lateral leaves (Fig. 2B–D, E, G) and both margins of the axillary and median leaves (Fig. 1B–D, F–H) broadly hyaline, as well as the apices of the lateral leaves acute (Fig. 1A, E; Fig. 2A, B, E, G).

**Figure 3.**
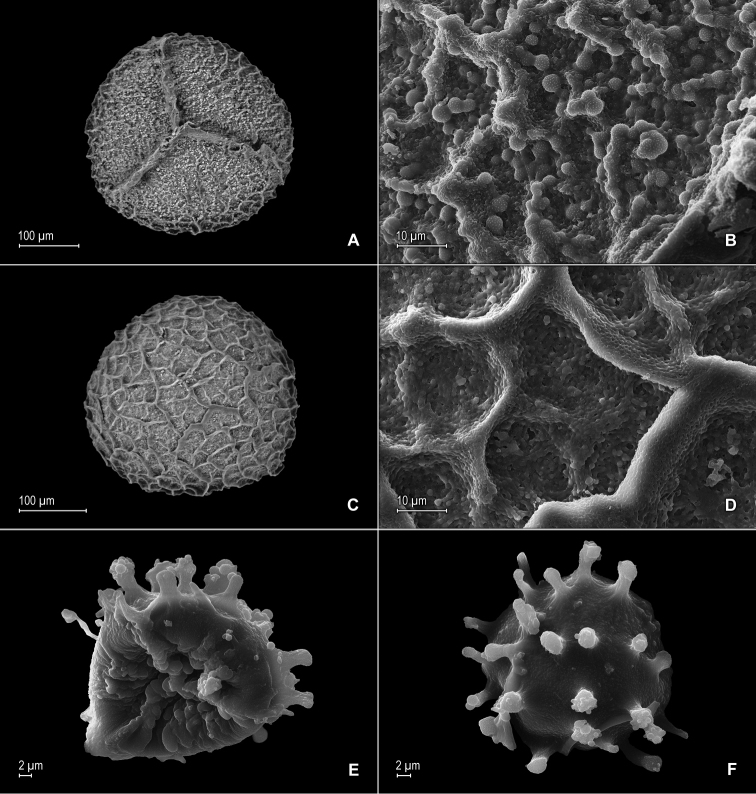
*Selaginella nanuzae* Valdespino. **A** Megaspore, proximal face **B** Close-up of megaspore, proximal face **C** Megaspore, distal face **D** Close-up of megaspore, distal face **E** Microspore, proximal face **F** Microspore, distal face. **A–F** taken from the holotype, *Salino et al. 7788* (PMA).

The two specimens cited here under *Selaginella nanuzae* were previously determined as *Selaginella contigua*, which as currently circumscribed is a morphologically variable species needing additional study. *Selaginella nanuzae* differs from typical *Selaginella contigua*, as lectotypified by Hirai and Prado (2000), by the characters given in the diagnosis. *Selaginella nanuzae* further differs from *Selaginella contigua* by its ovate-oblong or ovate (vs. oblong) lateral leaves with the apices acute (vs. truncate or broadly obtuse), and ovate or ovate-lanceolate (vs. ovate-lanceolate) axillary leaves with rounded bases (vs. rounded to cordate). In addition, *Selaginella nanuzae* has strongly imbricate leaves, while *Selaginella contigua* has leaves that are usually distant.

### Selaginella neospringiana

Taxon classificationPlantaeSelaginellalesSelaginellaceae

Valdespino sp. nov.

urn:lsid:ipni.org:names:77151572-1

Figures 4 , 5 , 6

#### Diagnosis.

*Selaginella neoespringiana* differs from *Selaginella vestiens* Baker by the median leaves elliptic (vs. ovate or ovate-lanceolate), each 0.6–0.8 × 0.3–0.45 mm (vs. 0.9–2.0 × 0.4–1.0 mm) with bases rounded (vs. bases truncate or with the inner bases truncate and the outer bases auriculate or bases oblique), hyaline margins 10–30 µm (vs. 100–180 µm) wide and long-ciliate (vs. dentate to short-ciliate), each cilia 80–180 µm (vs. 40–50 µm), the lateral leaves ovate to ovate-elliptic (vs. ovate-deltate) with the upper surfaces glabrous (vs. with submarginal prickle- or tooth-like projections along basiscopic halves), the basiscopic margins entire on proximal ⅔ and denticulate along distal ⅓ (vs. dentate throughout), and sporophylls short-ciliate (vs. dentate).

#### Type.

**BRAZIL**. Rio de Janeiro: Petrópolis, Morro de Cubiçado, Gularte area, [ca. 1650 m], 7 Jul 1879, *A. Glaziou 11723* (holotype: C!; isotypes: BM!, P [P01282486]-image!, P [P01282487]-image!, PMA! [PMA103270], US!).

#### Description.

*Plants* terrestrial (or epipetric?). *Stems* ascending to erect, stramineous, 3–5 cm long, 0.05–0.1 mm diam., non-articulate, flagelliform on branches, stoloniferous, 1- or 2-branched. *Rhizophores* axillary, borne on proximal ¼ of stems, filiform, 0.05 mm diam. *Leaves* heteromorphic throughout, membranaceous, both surfaces glabrous, upper surfaces green, lower surfaces silvery green. *Lateral leaves* spreading or slightly ascending, ovate to ovate-elliptic, 1.0–1.4 × 0.5–0.7 mm; bases rounded, acroscopic bases overlapping stems, basiscopic bases free from stems; acroscopic margins hyaline (more conspicuously so on lower surfaces), in a band 2–8 cells wide with the cells elongate, straight-walled and papillate parallel to margins, papillae in 1 or 2 rows over each cell lumen, long-ciliate throughout, basiscopic margins greenish or slightly hyaline in a band 1 or 2 cells wide with the cells rectangular, straight to sinuate-walled and papillate parallel to margins, papillae in 1–3 rows, long-ciliate throughout; apices cuspidate to short-acuminate, cusps 0.05 mm, variously tipped by 1–3 cilia; upper surfaces comprising quadrangular or rounded, sinuate-walled cells, many of these covered by 4–17 papillae, without idioblasts and with stomata along margins, lower surfaces comprising elongate, sinuate-walled cells, with few of these papillate and idioblast-like on both sides of the midribs, papillae in 2 rows over each cell lumen, with stomata in 2 rows along midribs. *Median leaves* distant, ascending, elliptic, 0.6–0.8 × 0.3–0.45 mm; bases rounded; margins hyaline in a band 2–6 cells wide, the cells elongate, straight-walled and papillate parallel to margins, papillae in 1 or 2 rows over each cell lumen, long-ciliate throughout; apices long-aristate, each arista 0.1–0.3 mm, denticulate distally on upper surfaces, tipped by 1 or 2 teeth; both surfaces without idioblasts, upper surfaces comprising quadrangular or rounded, sinuate-walled cells, many of these covered by 2–17 papillae, with stomata in 1 row along distal ¾ of the midribs and some along proximal ¼ of outer margins, lower surfaces comprising elongate, sinuate-walled cells, without stomata. *Axillary leaves* similar to lateral leaves. *Strobili* terminal on branch tips, quadrangular, 1.0–1.2 mm. *Sporophylls* monomorphic or the ventral ones slightly shorter, ascending, without a laminar flap, each with a slightly developed and glabrous keel along midribs, ovate to ovate-lanceolate, 0.7–1.2 × 0.5–0.8 mm; bases rounded; margins hyaline (this more obviously so on dorsal sporophylls), short-ciliate; apices acuminate to short-aristate, each acumen (arista) 0.05–0.1 mm, tipped by 1 or 2 teeth; *dorsal sporophylls* with upper surfaces green and cells as in median leaves, except for the half that overlaps the ventral sporophylls where the surfaces are hyaline with elongate, papillate, and slightly sinuate-walled cells, lower surfaces silvery green and comprising elongate, sinuate-walled cells; *ventral sporophylls* with both surfaces hyaline, comprising elongate, sinuate-walled cells. *Megasporangia* in 2 ventral rows; *megaspores* yellow, rugulate-reticulate on proximal faces with a slightly developed equatorial flange and perforate microstructure, reticulate on distal faces with echinulate and perforate microstructure (Fig. 6A–D), 185–265 µm. *Microsporangia* in 2 dorsal rows; *microspores* orange, rugulate on proximal faces with echinulate microstructure, echinulate or baculate on distal faces with echinulate microstructure (Fig. 6E–I), 24–30 µm.

**Figure 4.**
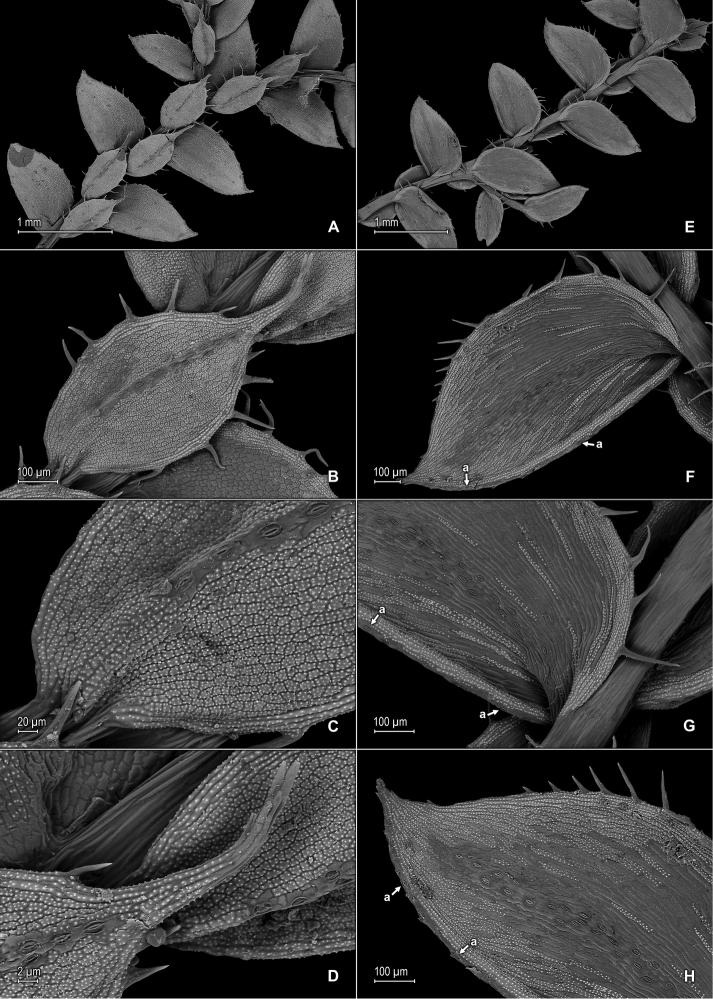
*Selaginella neospringiana* Valdespino. **A** Section of upper surface of stem **B** Upper surface of median leaf **C** Close-up of base and proximal portion of median leaf, upper surface **D** Close-up of distal portion and apex of median leaf, upper surface **E** Section of lower surface of stem **F** Lower surface of lateral leaf; note marginal stoma (a) **G** Close-up of proximal portion and base of lateral leaf, lower surface; note marginal stoma (a) on basiscopic margin and on outer margin of median leaf (far, lower right) **H** Close-up of distal portion and apex of lateral leaf, lower surface; note marginal stoma (a). **A–H** taken from the isotype, *Glaziou 11723* (PMA).

**Figure 5.**
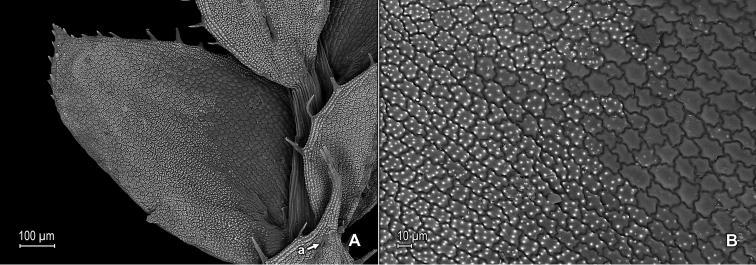
*Selaginella neospringiana* Valdespino. **A** Lateral leaf and portions of median leaves, upper surfaces; note papillae on cells lumen and stomata (a) along midrib of median leaf **B** Close-up of upper surface of lateral leaf; note papillae on cells lumen. **A–B** taken from the isotype, *Glaziou 11723* (PMA).

**Figure 6.**
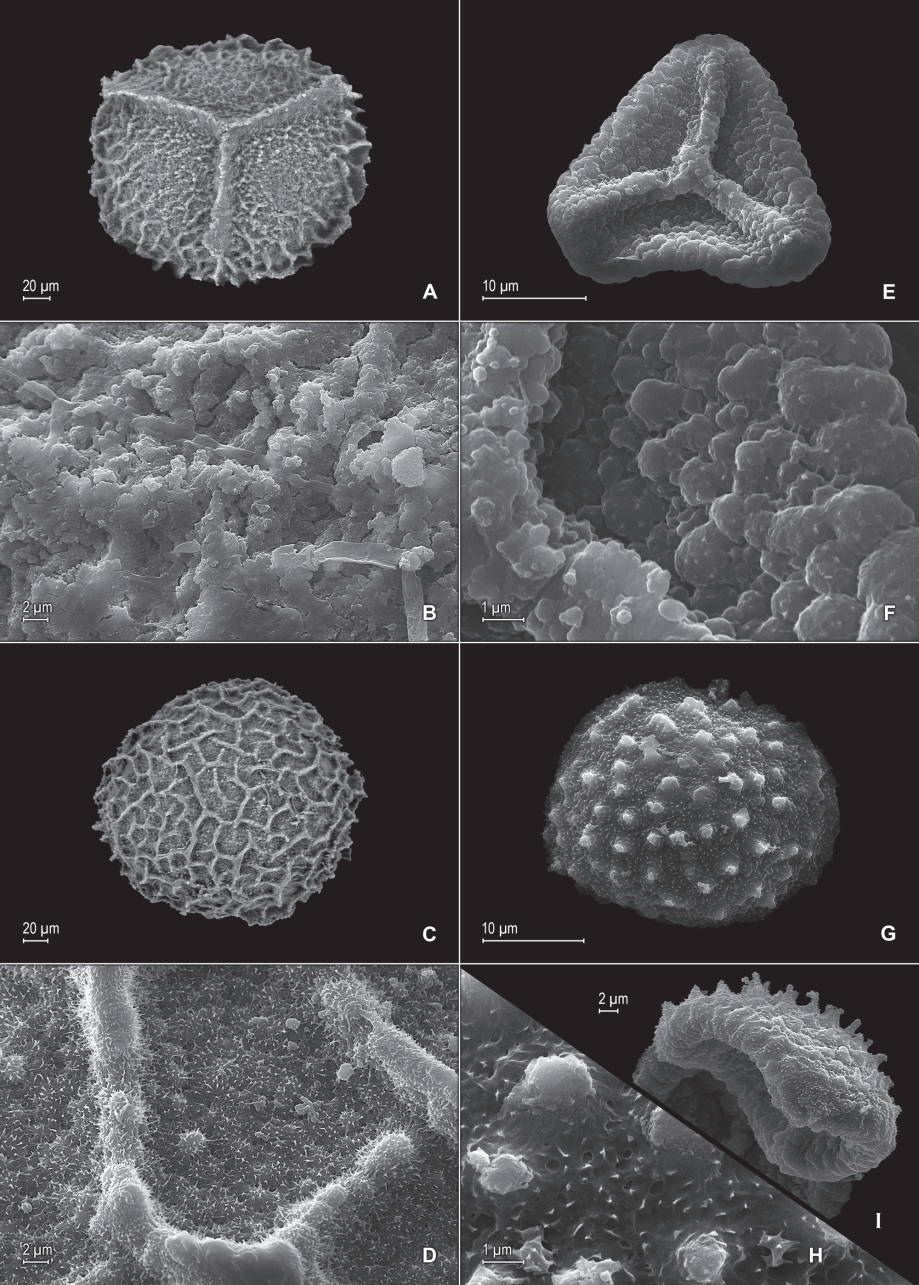
*Selaginella neospringiana* Valdespino. **A** Megaspore, proximal face **B** Close-up of megaspore, proximal face **C** Megaspore, distal face **D** Close-up of megaspore, distal face **E** Microspore, proximal face **F** Close-up of microspore, proximal face **G** Microspore, distal face **H** Close-up of microspore, distal face **I** Microspore, equatorial view. **A–I** taken from the isotype, *Glaziou 11723* (PMA).

#### Habitat and distribution.

*Selaginella neospringiana* is known only from the type collection made in Morro de Cubiçado, Petrópolis, Brazil. No information on the label exists as to its habitat, but in this region Campos de Altitude (highland fields or high-altitude fields) vegetation is common. The type collection was probably made at or around the peak of the Morro at ca. 1650 m and has some mosses associated with it; thus, *Selaginella neospringiana* may be terrestrial or epipetric.

#### Etymology.

The specific epithet honors Anton Friedrich Spring (1814–1872), a German physician and botanist who is the author of the only worldwide monograph of *Selaginella*. Spring described many species and proposed the first major classification of this ancient lycophyte genus. His contribution to our knowledge of *Selaginella* was significant. Therefore, it is fitting that this species collected at high elevations bears his name.

#### Conservation status.

As mentioned, *Selaginella neospringiana* is known only from the type collection made 135 years ago. It has apparently not been collected since, even though the area where it was gathered is visited by trekkers and adventurers. Taking this into account, this species is considered Endangered (En).

**Discussion.** Alston et al. (1981) cited the type of *Selaginella neospringiana* under *Selaginella vestiens*, but as pointed out by Valdespino et al. (2015) they are morphologically different and can be separated by the characters discussed under the diagnosis.

### Selaginella pellucidopunctata

Taxon classificationPlantaeSelaginellalesSelaginellaceae

Valdespino sp. nov.

urn:lsid:ipni.org:names:77151573-1

Figures 7 , 8 , 9

#### Diagnosis.

*Selaginella pellucidopunctata* differs from the similar *Selaginella muscosa* Spring by its median leaves elliptic or ovate-lanceolate (vs. broadly ovate to cordate), lateral leaves with the upper surfaces with few submarginal prickle- or tooth-like projections on basiscopic halves near basiscopic margins (vs. upper surfaces glabrous), with the acroscopic margins ciliate along proximal ½ (vs. denticulate throughout), axillary leaves ovate to broadly ovate (vs. broadly ovate or cordate), and megaspores deep yellow (vs. light yellow).

#### Type.

**BRAZIL**. Alagoas: Mpio. Ibateguara, Engenho Coimbra, Grota do Vargão, [ca. 09°00'02"S, 35°51'12"W], [ca. 500 m], 12 Nov 2001, *M. Oliveira 1094* (holotype: UFP! [UFP 39685]; isotype: PMA! [PMA103269]).

#### Description.

*Plants* terrestrial or epipetric. *Stems* ascending to erect, stramineous, 9–13 cm long, 0.4–0.7 mm diam., non-articulate, not flagelliform, shortly stoloniferous, 2–3-branched. *Rhizophores* axillary, borne on proximal ⅛–¼ of stems, filiform, 0.1–0.3 mm diam. *Leaves* heteromorphic throughout, membranaceous, both surfaces glabrous, upper surfaces green, lower surfaces silvery green. *Lateral leaves* spreading or slightly ascending, ovate to broadly ovate, 2.0–2.4 × 1.0–1.3 mm; bases rounded to almost semicordate, acroscopic bases strongly overlapping stems, basiscopic bases free from stems; acroscopic margins narrowly to broadly hyaline in a band 2–6 cells wide with the cells elongate, straight-walled and papillate parallel to margins, papillae in 1 row over each cell lumen, ciliate along proximal ½, otherwise dentate distally, basiscopic margins on upper surfaces greenish, comprising rounded or quadrangular, sinuate-walled cells, on lower surfaces narrowly to broadly hyaline in a band 2–4 cells wide with the cells as along acroscopic margins, denticulate throughout; apices acute to short-acuminate, each acumen 0.05–0.1 mm, variously tipped by 1–3 cilia; upper surfaces comprising rounded or quadrangular, sinuate-walled cells, some of these, particularly along submarginal and distal regions of the laminae, covered by 12–30 papillae, without idioblasts and with stomata along proximal ½ of basiscopic margins, lower surfaces comprising elongate, sinuate-walled cells, without conspicuous idioblasts (when viewed with stereomicroscope, EM) or these conspicuous (when viewed with SEM) and papillate on both sides of midribs, papillae in 1 or 2 rows over each cell lumen, with stomata in 2 or 3 rows along midribs and throughout acroscopic halves of the laminae. *Median leaves* distant, ascending, elliptic or ovate-lanceolate, 1.0–1.4 × 0.5–0.7 mm; bases rounded to slightly oblique; margins broadly hyaline in a band 1–7 cells wide, the cells elongate, straight-walled and papillate parallel to margins, papillae in 1 or 2 rows over each cell lumen, short-ciliate throughout or along proximal ⅔ and dentate distally or dentate throughout; apices long-aristate, each arista 0.5–0.7 mm, denticulate on upper surfaces, tipped by 1–3 cilia; both surfaces without idioblasts, upper surfaces comprising quadrangular or rounded, sinuate-walled cells, many of these covered by 7–20 papillae, with stomata in 4 rows along midribs, few stomata along proximal ¼ of outer margins, lower surfaces comprising elongate, sinuate-walled cells, without stomata. *Axillary leaves* similar to lateral leaves, except for both margins hyaline and long-ciliate along proximal ½, distally dentate. *Strobili* terminal on branch tips, dorsiventral, 0.2–1.0 cm. *Sporophylls* dimorphic; *dorsal sporophylls* spreading, with an adaxial laminar flap, each with a strongly developed and dentate keel along midribs, narrowly ovate to ovate-lanceolate, 1.5–1.8 × 0.5–0.7 mm; bases rounded; margins broadly hyaline, dentate to denticulate; apices acuminate, each acumen 0.1 or 0.2 mm with margins dentate and tipped by 2–4 teeth; upper surfaces green and cells as in median leaves, including many stomata, except for the half that overlaps the ventral sporophylls where the surfaces are hyaline with elongate, sinuate-walled cells, lower surfaces silvery green and comprising elongate, sinuate-walled cells; *ventral sporophylls* ascending, without a laminar flap, each with a slightly developed and dentate keel along midribs, ovate to ovate-lanceolate, 1.0–1.2 × 0.4–0.6 mm; bases rounded; margins broadly hyaline, dentate to denticulate; apices long-acuminate, each acumen 0.2 or 0.3 mm with margins dentate and tipped by 2–4 teeth; both surfaces hyaline, comprising elongate, straight-walled cells and papillate idioblasts. *Megasporangia* in 2 ventral rows; *megaspores* yellow, rugulate-reticulate on proximal faces with a prominent equatorial flange, reticulate on distal faces, with granulate-echinulate and perforate microstructure on both faces (Fig. 9A–D), 250–280 µm. *Microsporangia* in 2 dorsal rows; *microspores* orange, rugulate-echinulate on proximal faces, capitate on distal faces, with echinulate microstructure on both faces (Fig. 9E–H), 27–35 µm.

**Figure 7.**
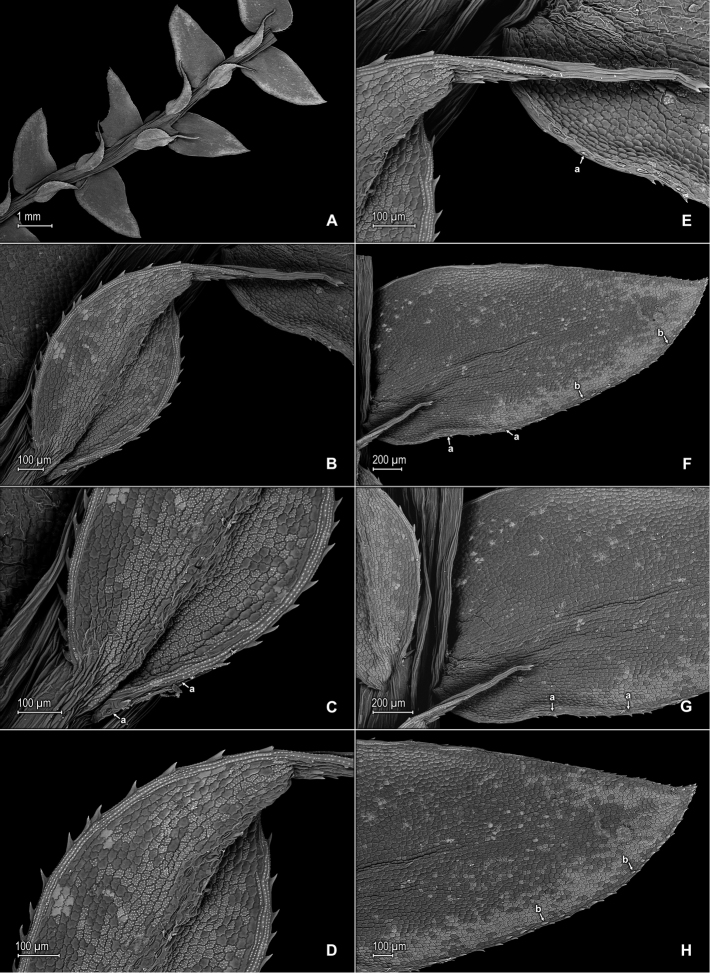
*Selaginella pellucidopunctata* Valdespino. **A** Section of upper surface of stem **B** Upper surface of median leaf **C** Close-up of base and proximal portion of median leaf, upper surface; note marginal stoma (a) on outer margin **D** Close-up of median leaf, distal region, upper surface **E** Close-up of apex of median leaf, upper and lower surfaces; note marginal stoma (a) on basiscopic margin of lateral leaf, upper surface **F** Upper surface of lateral leaf; note marginal stoma (a) on basiscopic margin and submarginal tooth (b) **G** Close-up of base and proximal portion of lateral leaf, upper surface; note marginal stoma (a) on basiscopic margin **H** Close-up of distal portion and apex of lateral leaf, upper surface; note submarginal tooth (b). **A–H** taken from the isotype, *Oliveira 1094* (PMA).

**Figure 8.**
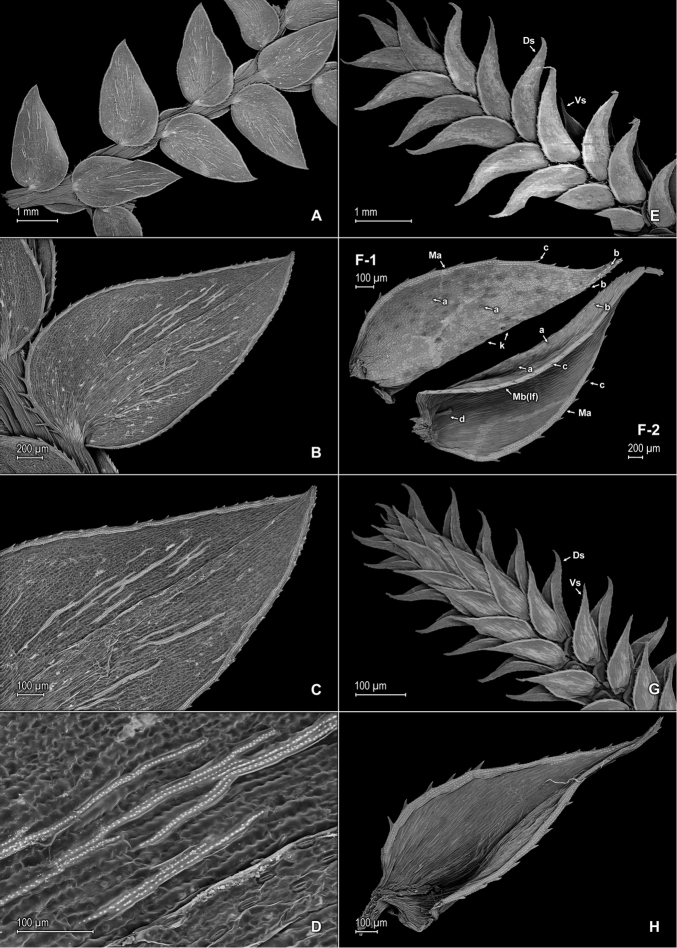
*Selaginella pellucidopunctata* Valdespino. **A** Section of lower surface of stem **B** Lower surface of lateral leaf **C** Close-up of distal portion and apex of lateral leaf, lower surface; note elongate, straight-walled, and papillate cells (idioblasts) **D** Close-up of lateral leaf, lower surface; note elongate, straight-walled, and papillate cells (idioblasts) **E** Strobilus, upper surface; note dorsal (Ds) and ventral (Vs) sporophyll **F** Dorsal sporophylls, adaxial- (F-1) and abaxial surfaces (F-2); note acroscopic margin (Ma) and basiscopic margin (Mb), this usually referred to as laminar flap (lf), stomata (a) throughout lamina and midrib, as well as tooth-like projections (b) on midrib and lamina, abaxial (upper) surface, marginal tooth projections (c), and ligule (d) **G** Strobilus, lower surface; note dorsal- (Ds) and ventral (Vs) sporophyll **H** Ventral sporophyll, adaxial (lower) surface. **A–H** taken from the isotype, *Oliveira 1094* (PMA).

**Figure 9.**
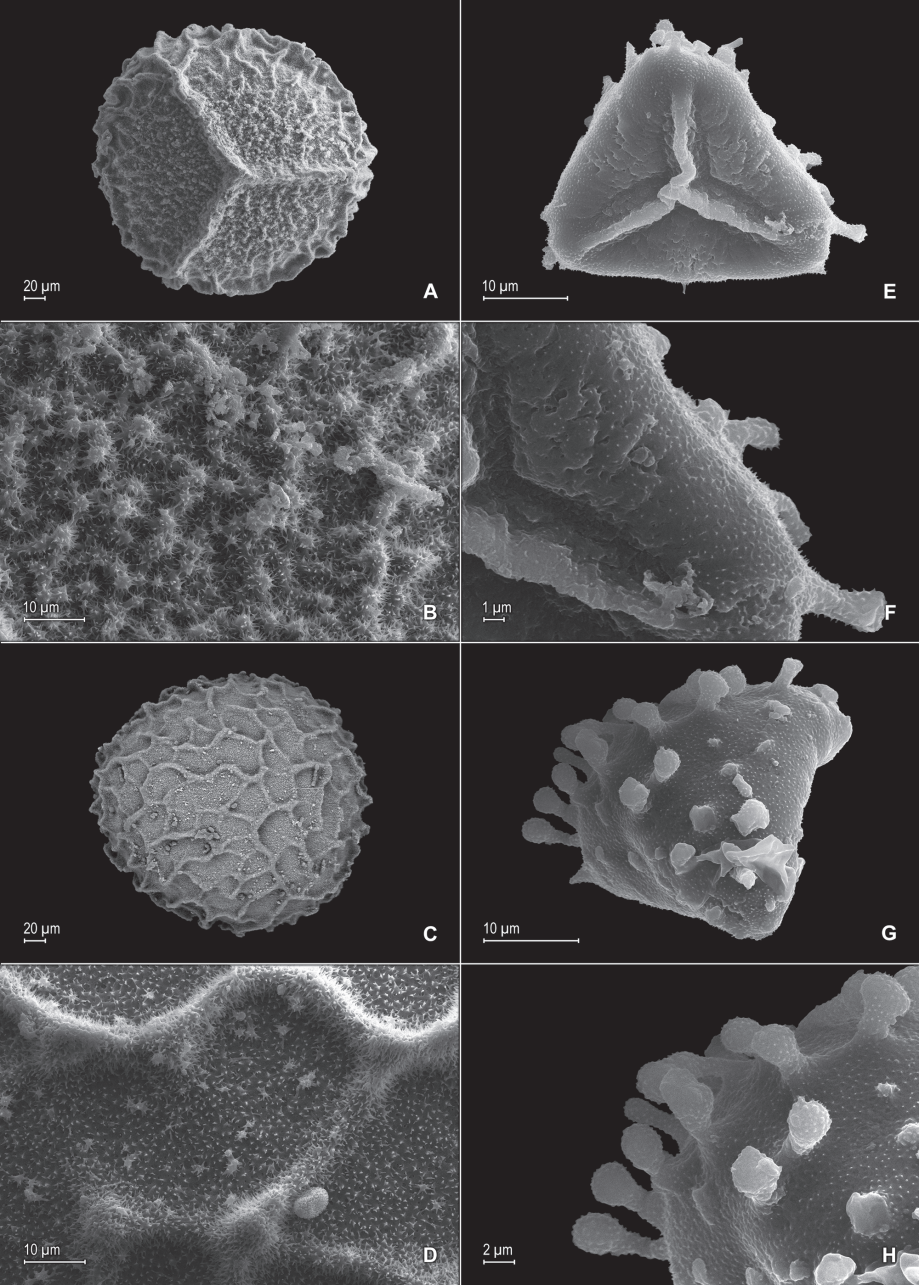
*Selaginella pellucidopunctata* Valdespino. **A** Megaspore, proximal face **B** Close-up of megaspore, proximal face **C** Megaspore, distal face **D** Close-up of megaspore, distal face **E** Microspore, proximal face **F** Close-up of microspore, proximal face **G** Microspore, distal face **H** Close-up of microspore, distal face. **A**–**H** taken from the isotype, *Oliveira 1094* (PMA).

#### Habitat and distribution.

*Selaginella pellucidopunctata* grows along stream banks or near bushes in flagstones of inselbergs in the Atlantic semi-deciduous forest vegetation at 300–650 m. It is known only from the states of Alagoas and Pernambuco in Brazil.

#### Etymology.

The specific epithet is derived from the Latin *pellucidus*, meaning translucent, and *punctatus*, dotted; this alludes to many, conspicuous stomata on the greenish upper surfaces of dorsal sporophylls that resemble translucent dots.

#### Conservation status.

Given that few collections are available, I cannot provide a definitive conservation status assessment of *Selaginella pellucidopunctata*. Nevertheless, this species occurs in one of the most critically endangered ecoregions of Brazil, the Atlantic Pernambuco interior forest of Northeaster, which is highly deforested with only five percent of the original vegetation present (WWF 2015). Therefore, *Selaginella pellucidopunctata* is preliminarily considered Endangered (En).

#### Additional specimens examined

**(paratypes). BRAZIL**. **Alagoas**: Mpio. Ibateguara, Usina Serra Grande, Engenho Coimbra, [ca. 09°00'02"S, 35°51'12"S], [ca. 500 m], 15 Oct 2003, *Pietrobom et al. 5637* (UFP, PMA); Mpio. São José da Lage, Usina Serra Grande, 08°59'42.4"S, 36°07'28.9"W, ca. 380–415 m, 8 Feb 2001, *Pietrobom & Santiago 4807* (UFP, PMA). **Pernambuco**: Mpio. Jaqueira, Usina, Colônia, 08°04'15"S, 35°50'13"W, ca. 650 m, 17 Oct 2001, *Lopes* & *Pietrobom 350* (RB-image, UFP-n.v.), 08°43'21.1"S, 35°50'22.1"W, ca. 545 m, 20 May 2002, *Lopes, 593* (RB-image, UFP-n.v.); Mpio. Timbaúba, Complexo da Serra do Mascarnhas, Usina Cruangi, Engenho Água Azul, ca. 07°36'31.5"S, 35°22'42.9"W, ca. 304–394 m, 13 Nov 2000, *Pietrobom et al. 4646* (UFP, PMA).

#### Discussion.

Among Brazilian *Selaginella*, *Selaginella pellucidopunctata* most resembles *Selaginella muscosa*. They differ most noticeably by the characters of the median leaf shape and the projections on the upper surfaces and margins of the lateral leaves, as discussed in the Diagnosis. In addition, the leaf surfaces of *Selaginella pellucidopunctata*, when viewed with EM, seem to lack (vs. exhibit) conspicuous idioblasts. However, idioblasts are seen on SEM images of the lower surfaces of lateral leaves of *Selaginella pellucidopunctata*.

One specimen, *Lopes* & *Pietrobom 350* at RB ([RB 375875]–image!) is identified as *Selaginella arenaria* Baker, a synonym of *Selaginella brevifolia* Baker (Valdespino 2015c), which is a species characterized by its lateral leaves with the upper surfaces hispidulous with prickle- or tooth-like projections usually found submarginally, marginally, and apically along the basiscopic halves of the laminae, and with conspicuous, straw-colored midribs. Another specimen, *Lopes 593* at RB ([RB 375877]-image!) is identified as *Selaginella tenuissima* Fée, which is a creeping to prostrate species with usually cordate median leaves.

### Selaginella stomatoloma

Taxon classificationPlantaeSelaginellalesSelaginellaceae

Valdespino sp. nov.

urn:lsid:ipni.org:names:77151574-1

Figure 10

#### Diagnosis.

*Selaginella stomatoloma* differs from the recently described *Selaginella saltuicola* Valdespino by having the upper surfaces of the leaves with glabrous (vs. papillate) cells, the lateral leaf lower surfaces without (vs. with) papillate idioblasts and with marginal cells glabrous (vs. papillate), and acute (vs. rounded to broadly acute) apices, the median leaf margins comprising slightly elongate and glabrous (vs. strongly elongate and papillate) cells, these denticulate (vs. entire), with short-acuminate (vs. acute) apices, and upper surfaces with few submedial and submarginal stomata (vs. stomata throughout laminae).

#### Type.

**BRAZIL**. Pará: Canaã dos Carajás, S11D, UTM-Zone 22M: 9293819 575625 [06°23'08"S, 50°18'58.24"W], 31 Aug 2010, *T.E. Almeida et al. 2518* (holotype: PMA! [PMA103369]; isotype: BHCB [BHCB142524]-n.v.).

#### Description.

*Plants* epipetric. *Stems* decumbent to ascending, stramineous, 3–6 cm long, 0.2–0.5 mm diam., non-articulate, not flagelliform, stoloniferous, 1- or 2-branched. *Rhizophores* axillary, borne on proximal ¼–¾ of stems, filiform, 0.05 or 0.15 mm diam. *Leaves* heteromorphic throughout, membranaceous, both surfaces glabrous, upper surfaces green, lower surfaces silvery green. *Lateral leaves* spreading to ascending near branches and stem apices, ovate-elliptic to ovate-oblong, 1.5–2.0 × 0.8–0.9 mm; bases rounded, acroscopic bases slightly overlapping stems, basiscopic bases free from stems; margins greenish with the cells quadrangular, sinuate-walled, glabrous, mostly entire and denticulate near apices or denticulate throughout; apices acute and variously tipped by 1 or 2 teeth; both surfaces without idioblasts, upper surfaces comprising quadrangular to rounded, sinuate-walled, glabrous cells, with stomata submarginal, particularly on distal ½ and throughout margins, lower surfaces comprising elongate, sinuate-walled, glabrous cells, with stomata in 3–5 rows along midribs and throughout acroscopic halves of the laminae. *Median leaves* distant or imbricate near branches and stem apices, ascending, ovate-elliptic or elliptic, 0.7–1.1 × 0.3–0.6 mm; bases rounded to slightly oblique; margins greenish or narrowly hyaline in a band 2 or 3 cells wide, the cells slightly elongate, straight-walled and glabrous parallel to margins, denticulate throughout; apices short-acuminate, each acumen 0.05–1.05 mm, denticulate on upper surfaces, tipped by 1 or 2 teeth; both surfaces without idioblasts, upper surfaces comprising quadrangular to rounded, sinuate-walled, glabrous cells, with stomata in 1–3 rows along midribs, with a few submedial and submarginal and throughout margins, lower surfaces comprising elongate, sinuate-walled cells, without stomata. *Axillary leaves* similar to lateral leaves. *Strobili* terminal on branch tips, quadrangular, 1.5–6.0 mm. *Sporophylls* monomorphic, without a laminar flap, each with a strongly developed and seemingly entire or denticulate keel along midribs, ovate or the ventral ones broadly ovate, 0.9–1.2 × 0.4–0.7 mm; bases rounded; margins narrowly hyaline (less so on ventral sporophylls), denticulate; apices acute to short-acuminate, each acumen 0.05–0.1 mm with margins dentate and tipped by 1–2 teeth; *dorsal sporophylls* with upper surfaces green and cells as in median leaves, including stomata, lower surfaces greenish and comprising elongate, sinuate-walled cells; *ventral sporophylls* with both surfaces greenish, comprising elongate, sinuate-walled cells. *Megasporangia* in 2 ventral rows or few and intermixed with microsporangia; *megaspores* yellow, mostly immature, rugulate-reticulate on proximal faces, reticulate on distal faces, microstructure not examined, ca. 200 µm. *Microsporangia* in 2 dorsal rows and on ventral rows or also in axil of median leaves immediately below strobili; *microspores* light orange, ornamentation and diameter not determined.

#### Habitat and distribution.

*Selaginella stomatoloma* is an epipetric species known only from the state of Pará in Brazil. It grows in dense lowland to premontane wet forests, probably at 200–800 m.

#### Etymology.

The specific epithet is derived from the Greek *stoma*, meaning mouth, and *loma*, fringe or border; together these refer to the many stomata found on leaf margins.

#### Conservation status.

*Selaginella stomatoloma* is known only from three collections made in de Carajás National Forest, Brazil. This area is threatened by deforestation due to cattle ranching and large-scale mining (Pinheiro et al. 2012). Therefore, *Selaginella stomatoloma* is considered Vulnerable (VU).

#### Additional specimens examined

**(paratypes). BRAZIL**. **Pará**: Canaã dos Carajás, S11D, [ca. 06°23'08"S, 50°18'58.24"W], 23 May 2012, *Salino et al. 15284* (BHCB, PMA), 27 Aug 2012, *Salino et al. 15492* (BHCB, PMA).

#### Discussion.

**Figure 10.**
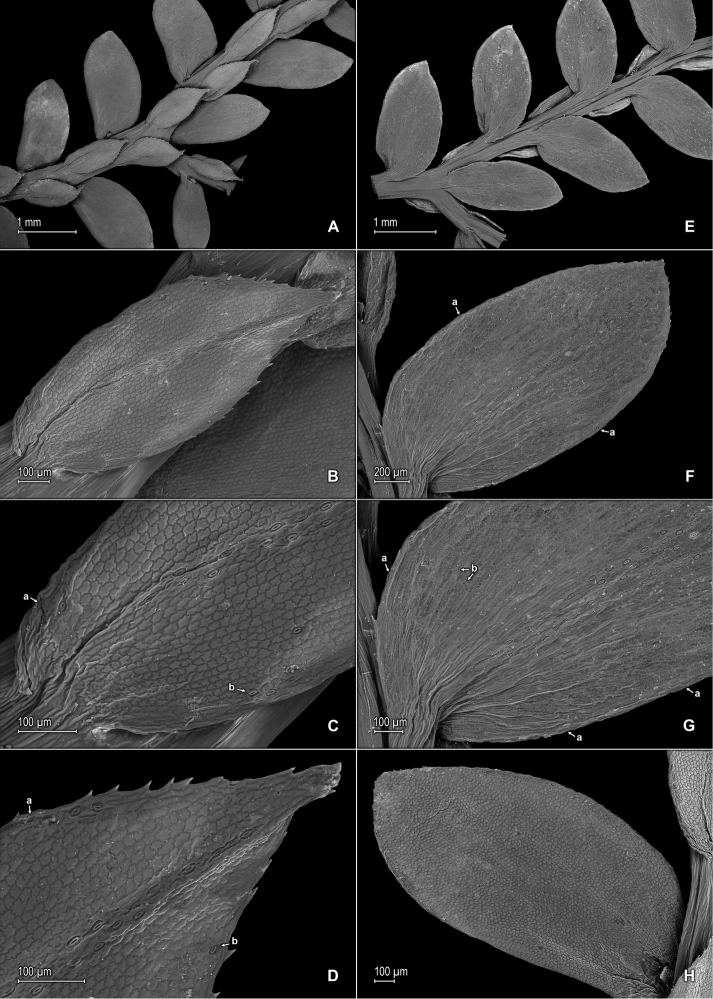
*Selaginella stomatoloma* Valdespino. **A** Section of upper surface of stem **B** Upper surface of median leaf **C** Close-up of base and proximal portion of median leaf, upper surface; note marginal (a) and submarginal stomata (b) **D** Close-up of proximal portion and apex of median leaf, upper surface; note marginal (a) and submarginal (b) stomata **E** Section of lower surface of stem **F** Lower surface of lateral leaf; note marginal stomata (a) **G** Close-up of base and proximal portion of lateral leaf, lower surface; note marginal (a) and submarginal (b) stomata **H** Upper surface of lateral leaf. **A–H** taken from the holotype, *Almeida et al. 2518* (PMA).

The minute plant size, stomata along leaf margins (Fig. 10), and microsporangia in the axils of the median leaves near the strobili are features that *Selaginella stomatoloma* shares with *Selaginella saltuicola*. They differ, however, by the characters discussed in the diagnosis.

The presence of stomata along leaf margins seems to be a more common feature in *Selaginella* than previously realized. This distribution was shown to occur in several species by, for example, Harvey-Gibson (1897), Schulz et al. (2010), Youguang and Tan (2013), and Valdespino et al. (2015), and are described here in *Selaginella nanuzae* (Fig. 1, 2), *Selaginella neospringiana* (Fig. 4), *Selaginella pellucidopunctata* (Fig. 7, 8), *Selaginella stomatoloma* (Fig. 10), and *Selaginella trygonoides* (Fig. 12), as well as being present on the outer margin of median leaves of *Selaginella roraimensis* Baker (see Fig. 11C in Alston et al. 1981: 284, shown as *Selaginella scintillata* Alston). Youguang and Tan (2013) hypothesized that leaf marginal stomata were non-functional; however, the widespread occurrence of this feature in morphologically distinct species from diverse regions of the world indicate the contrary, particularly when one considers their fundamental role in plant physiology. It seems evolutionarily inefficient for *Selaginella* to develop leaf marginal stomata (as well as on other parts of the lamina), besides those along the midribs, if they were not active in playing key roles in plant photosynthesis. In the absence of experimental evidence to the contrary, they are here considered functional. Nevertheless, these competing explanations for the distribution and physiological importance of stomata on *Selaginella* highlight the need for detailed experimental studies, to address functional issues.

**Figure 11.**
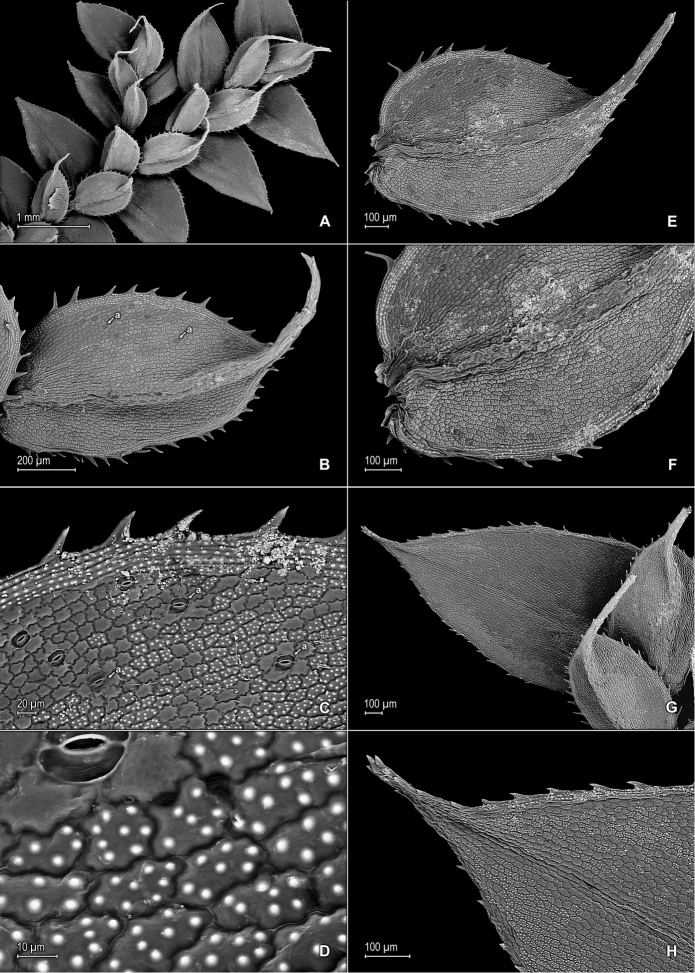
*Selaginella trygonoides* Valdespino. **A** Section of upper surface of stem **B** Upper surface of median leaf; note submarginal stomata (a) on inner half of the lamina **C** Close-up of inner margin of median leaf, upper surface; note submarginal stomata (a) **D** Close-up of median leaf upper surface; note papillae on cell lumen **E** Upper surface of median leaf; note submarginal stomata on inner half of the lamina **F** Close-up of base, portion of inner margin and inner half of lamina (note stomata), and portion of outer margin and outer half of lamina of median leaf, upper surface **G** Upper surface of lateral leaf and portion of median leaves, upper surface **H** Close-up of distal portion and apex of lateral leaf, upper surface. **A–H** taken from the holotype, *Almeida et al. 1994* (PMA).

**Figure 12.**
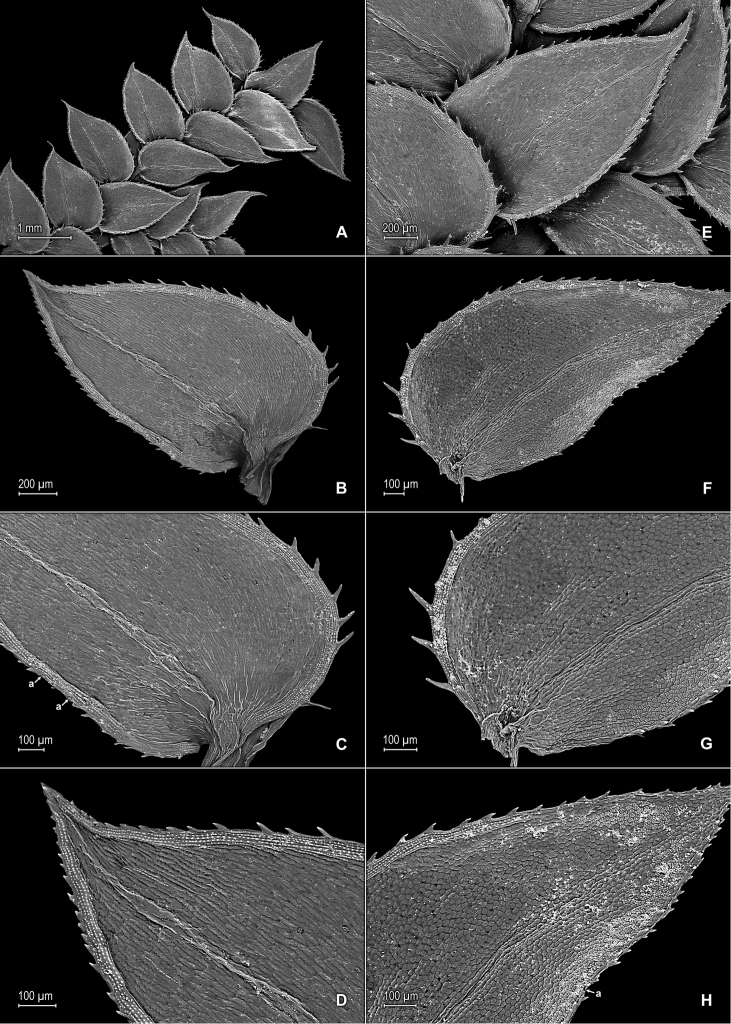
*Selaginella trygonoides* Valdespino. **A** Section of lower surface of stem **B** Lower surface of lateral leaf **C** Close-up of base and proximal portion of lateral leaf, lower surface; note marginal stomata (a) **D** Close-up of distal portion and apex of lateral leaf, lower surface **E** Lower surface of axillary leaf and portions of lateral leaves **F** Upper surface of lateral leaf **G** Close-up of base and proximal portion of lateral leaf, upper surface **H** Close-up of distal portion and apex of lateral leaf, upper surface; note marginal stoma (a). **A–H** taken from the holotype, *Almeida et al. 1994* (PMA).

### Selaginella trygonoides

Taxon classificationPlantaeSelaginellalesSelaginellaceae

Valdespino sp. nov.

urn:lsid:ipni.org:names:77151575-1

Figures 11 , 12

#### Diagnosis.

*Selaginella trygonoides* differs from the similar *Selaginella glazioviana* Hieron. by having the upper surfaces of the leaves dull (vs. shiny due to thick waxy deposits covering cell walls), median leaf margins short-ciliate (vs. entire to denticulate) with the arista ½ (vs. usually ¼–⅓) the length of the lamina, and lateral leaves acuminate (vs. broadly acute to acute).

#### Type.

**BRAZIL**. Minas Gerais: Serra do Azeite, Pocrane, 19°30'12"S, 41°37'47"W, 300 m, 1 Jun 2009, *T.E. Almeida, D.T. Souza & M.M.T. Cota 1994* (holotype: PMA! [PMA103371]; isotype: BHCB [BHCB130573]-n.v.).

#### Description.

*Plants* terrestrial or epipetric. *Stems* ascending to erect, stramineous, 3–5 cm long, 0.2–0.5 mm diam., non-articulate, not flagelliform or stoloniferous, 1- or 2-branched. *Rhizophores* axillary, borne on proximal ¼–½ of stems, filiform, 0.1 or 0.2 mm diam. *Leaves* heteromorphic throughout, chartaceous, both surfaces glabrous, upper surfaces green, lower surfaces silvery green. *Lateral leaves* spreading or ascending, ovate-deltate or ovate-elliptic, 1.5–2.2 × 0.8–1.1 mm; bases rounded, acroscopic bases strongly overlapping stems, basiscopic bases free from stems; acroscopic margins broadly hyaline in a band 2–6 cells wide with the cells elongate, straight-walled, and papillate parallel to margins, papillae in 1 row over each cell lumen, short-ciliate along proximal ½, otherwise dentate distally; basiscopic margins on upper surfaces greenish comprising quadrangular, sinuate-walled, glabrous and papillate cells, on lower surfaces broadly hyaline in a band 2–6 cells wide with the cells as along acroscopic margins, dentate to denticulate throughout; apices acuminate, each acumen 0.1 or 0.2 mm, variously tipped by 1–3 teeth; upper surfaces comprising rounded or quadrangular, sinuate-walled cells, some of these covered by 3–11 papillae, without idioblasts or stomata, lower surfaces comprising elongate, sinuate-walled cells, with few of these papillate and idioblast-like on both sides of the midribs, papillae in 1 row over each cell lumen, with stomata in 2 or 3 rows along midribs and throughout acroscopic half of the lamina. *Median leaves* imbricate or distant, ascending, broadly ovate-elliptic, 0.8–1.2 × 0.5–0.9 mm; bases rounded to slightly oblique; margins broadly hyaline in a band 3–7 cells wide, the cells elongate, straight-walled and papillate parallel to margins, papillae in 1 row over each cell lumen, short-ciliate throughout or along proximal ⅔ and dentate distally; apices long-aristate, each arista 0.4–0.6 mm, denticulate on upper surfaces, tipped by 1–3 teeth; both surfaces without idioblasts, upper surfaces comprising rounded or quadrangular, sinuate-walled cells, many of these covered by 4–14 papillae, with stomata in 4 rows along midribs, few scattered throughout inner halves and on margins of outer halves of the laminae, lower surfaces comprising elongate, sinuate-walled cells, without stomata. *Axillary leaves* similar to lateral leaves, except for both margins hyaline and short-ciliate along proximal ½ and distally dentate. *Strobili* terminal on branch tips, loosely quadrangular, 0.5–1.0 cm. *Sporophylls* monomorphic to subdimorphic, without a laminar flap, each with a strongly developed and dentate keel along midribs, ovate to ovate-lanceolate, 0.9–1.2 × 0.4–0.6 mm; bases rounded; margins broadly hyaline (this more obviously so on dorsal sporophylls), short-ciliate or dentate on ventral sporophylls; apices acuminate to short-aristate, each acumen (arista) 0.2–0.4 mm with margins dentate and tipped by 1 or 2 teeth; *dorsal sporophylls* with upper surfaces green and cells as in median leaves, including stomata, lower surfaces silvery green and comprising elongate, sinuate-walled cells; *ventral sporophylls* with both surfaces hyaline, comprising elongate, sinuate-walled cells. *Megasporangia* in 2 ventral rows; *megaspores* yellow, mostly immature, rugulate-reticulate on proximal faces with a prominent equatorial flange, reticulate on distal faces, microstructure not determined, ca. 200 µm. *Microsporangia* in 2 dorsal rows; *microspores* light orange, ornamentation and microstructure not determined.

#### Habitat and distribution.

*Selaginella trygonoides* is known only from the state of Minas Gerais, Brazil, where it may be endemic. It grows on creek banks in Gallery forests or Atlantic semi-deciduous forests vegetation at 185–300 m.

#### Etymology.

The specific epithet is derived from the Latin “*trigon*/*trygonus*”, meaning stingray; it alludes to the shape of the median leaf, which resembles these marine fish.

#### Conservation status.

*Selaginella trygonoides* is known only from two collections made within or nearby populated areas; most likely it is subjected to anthropomorphic pressures. Thus, I tentatively consider it Vulnerable (VU).

#### Additional specimen examined

**(paratype). BRAZIL**. **Minas Gerais**: Santa Rita do Itueto, Região da Cachoeira do Pontão, 19°24'52"S, 41°22'45"W, 185 m, 27 May 2009, *Almeida et al. 1960* (BHCB-n.v., PMA).

#### Discussion.

*Selaginella trygonoides* is morphologically close to *Selaginella glazioviana*, but it is distinguished from the latter by the characters of leaf surfaces and apex type, as well as median leaf marginal projections, as discussed in the diagnosis. In addition, *Selaginella trygonoides* grows in lowland vegetation at 185–300 m, whereas *Selaginella glazioviana* is found in montane vegetation at 900–1600 m. *Selaginella glazioviana* was thought to be conspecific with *Selaginella erectifolia* Spring by Alston (1936), Reed (1965–1966), and Alston et al. (1981), but I consider these taxa to be distinct species (see discussion under *Selaginella glazioviana*). *Selaginella trygonoides* differs from *Selaginella erectifolia* by its lateral leaves ovate-deltate (vs. ovate) with the acroscopic margins short-ciliate along proximal ½ (vs. dentate) and apices acuminate (vs. acute), the median leaves with short-ciliate (vs. dentate) margins, apices long-aristate (vs. short-aristate) with each arista ½ (vs. ¼) the length of the lamina, the upper surface with (vs. lacking) stomata on the inner half of the leaf lamina and some cells with the lumen covered by 4–8 (vs. 14–25) papillae.

Another collection, *Almeida & Souza 336* (PMA!), gathered in the same general locality of *Selaginella trygonoides*, is provisionally referred to *Selaginella decomposita* Spring. This collection is similar to *Selaginella trygonoides* in having lateral leaves acuminate and median leaves aristate; however it differs by its prostrate to ascending habit, stems to 3-branched, dorsal and ventro-axillary rhizophores, shiny leaves, median leaves dentate throughout with prominent outer bases, lateral leaves ovate-oblong to oblong, and axillary leaves ovate-lanceolate to ovate-elliptic.

### New distribution records

#### Selaginella beitelii

Taxon classificationPlantaeSelaginellalesSelaginellaceae

A.R. Sm.

Selaginella beitelii A.R. Sm., Ann. Missouri Bot. Gard. 77: 264. 1990. — Type. Venezuela. Amazonas: Cerro de la Neblina, Camp 7, 5.1 km NE Pico Phelps, along Caño Gardner, 01°50'40"N, 65°58'10"W, 1735 m, 30 Jan 1985, *J. Beitel 85079* (holotype: UC! [UC1551881]; isotype: NY! [NY00144076]).

##### Additional specimen examined.

**BRAZIL**. **Amazonas**: São Gabriel da Cachoeira, Parque Nacional do Pico da Neblina, trail to Cachoeira Anta, between Camp Lajero and Marco 5 of the borderline between Brazil and Venezuela, 00°49'08"N, 65°58'01"W, 2272 m, 30 Dec 2004, *Carvalho et al. 353* (INPA-n.v., PMA).

##### Discussion.

*Selaginella beitelii* was considered endemic to Sierra (Cerro) de la Neblina in Venezuela (Smith 1990). It is now known from the Brazilian side of this mountain. It is terrestrial or epiphytic and characterized by having axillary rhizophores, median leaves broadly ovate to semicordate with the outer base prominently lobed and the inner base oblique, the margins narrowly hyaline (more so on inner ones) and denticulate, and the lamina abruptly tapering towards the apex, the lateral leaves broadly ovate to semicordate with the acroscopic half almost twice as wide as the basiscopic half, the acroscopic margin narrowly hyaline to greenish, the basiscopic margin greenish, and both margins denticulate to entire.

#### Selaginella cabrerensis

Taxon classificationPlantaeSelaginellalesSelaginellaceae

Hieron.

Selaginella cabrerensis Hieron., Hedwigia 43: 29. 1904. — Type. Colombia. Tolima: Río Cabrera, 500–1000 m, Jan 1886, *F.C. Lehmann 6406* (holotype: B! [B 20 0095103]; isotypes: K! [K000589282], US! [US00135715]).
Selaginella arroyoana M. Kessler & A.R. Sm., Edinburgh J. Bot. 63: 87. 2006. — Type: Bolivia. Depto. Santa Cruz: Prov. Velazco, Parque Nacional Noel Kempff M., Campamento Las Gamas, 14°48'11"S, 60°23'35"W, 900 m, 30 Mar 1993, *L. Arroyo & K. Keil, 202* (holotype: LPB-n.v.; isotypes: MO!, NY! [NY01104443], UC [UC 1613683]-image!, USZ-n.v.).
Selaginella chiquitana M. Kessler, A.R. Sm. & M. Lehnert, Edinburgh J. Bot. 63: 91. 2006. — Type: Bolivia. Depto. Santa Cruz: Prov. Chiquitos, Serranía de Santiagos, en la mesa de Arco de Piedra, 18°20'S, 59°35'W, 800 m, 23 Feb 2003, *M. Lehnert 642* (holotype: UC [UC 1717964]-image!; isotypes: GOET-n.v., LPB-n.v.).

##### Additional specimens examined.

**COLOMBIA. Cundinamarca**: Pandi, 900 m, 9 Feb 1876, *André 1817* (BM, NY), Mpio. Pandi, near Río Sumapaz, 800 m, 27 Sep 1975, *Acosta-Arteaga 1020* (COL). **Meta**: Sierra de la Macarena, 900 m, 6 Feb 1950, *Philipson 2388* (BM, COL). **Tolima**: Chicoral, 450 m, 4 Mar 1949, *Haught 6333* (BM), near Río Coello, 13 May 1949, *Haught 6439* (BM); Valle de San Juan, near Minas del Sapo, 1400 m, 10 Jun 1966, *Echeverry 1303* (COL). **FRENCH GUIANA**. Extension nord-ouest des Petites Montagnes Tortue, 1 km S of RN 2 and 16.5 km WNW de Régina (Guyana), 04°20'N, 52°16'W, 90 m, 14 Mar 2006, *Boudrie 4177* (NY). **BOLIVIA**. **Depto. Santa Cruz**: Prov. Velazco, Parque Nacional Noel Kempff M., Campamento La Torre, 13°39'14"S, 60°49'50"W, 250 m, 21 Nov 1993, *Killen et al. 6200* (MO, UC), Serranía de Caparuch, 13°39'00"S, 60°54'00"W, 850 m, 21 Apr 1993, *Killen et al. 5433* (MO, UC). **BRAZIL**. **Goiás**: Mpio. Caldas Novas, Rodovia GO-413 (GO-15) Caldas Novas-Mazagão, ca. 18 km de Caldas Novas, margem do Rio, 17°44'S, 48°36'W, ca. 550 m, 23 Jan 1996, *Pietrobom 2648* (PMA, UFP); Côrrego Itaquera, ca. 30 km N of Formosa, 850 m, 2 May 1966, *Irwin et al. 15552* (NY); Mpio. of Pirenópolis, Parque Estadual da Serra dos Pireneus, 15°51'13"S, 48°51'30"W, 4 Feb 2011, *da Silva et al. 7295* (BHCB), ca. 15 km NE of Corumbá de Goiás, 1250–1300 m, 14 May 1973, *Anderson 10255* (NY), ca. 10 km NE of Corumbá de Goiás, Río Corumbá, 1050 m, 15 May 1973, *Anderson 10341* (K, NY, UC). **Mato Grosso**: ca. 5 km N of Barra do Garças, 500 m, 7 May 1973, *Anderson 9899* (NY); First Igarapé on road to Cuiaba, 600 m, 23 Oct 1973, *Prance et al. 19335* (NY, UC); Chapada dos Guimarães, 13 Feb 1975, *Hatschbach et al. 36114* (NY), Veu de Novia, 720 m, 25 Oct 1973, *Prance et al. 19392* (NY). **Mato Grosso do Sul**: Mpio. Costa Rica, Rodovia MS Cassilândia-Alto Araguaia, Povoado Laje, Rio Laje, [18°47'S, 54°14'W], 5 Sep 1993, *Rodrigues & Pietrobom 551* (PMA, UFP), 18°47'S, 54°14'W, ca. 715 m, 5 Sep 1993, *Rodrigues & Pietrobom 563* (PMA, UFP), Rodovia MS-306, Cachoeira no Rio Laje, 18°47'S, 54°13'W, 500 m, ca. 500 m, 20 Feb 1996, *Nonato et al. 262* (PMA, UFP); Mpio. Rio Verde do Mato Grosso, Rodovia Sete Quedas-Rio Negro, ca. 30 km da cidade, Fazenda Mirante, Cachoeira do Cervo, ca. 18°55'S, 54°53'W, ca 370 m, 7 Sep 1993, *Rodrigues & Pietrobom 660* (PMA, UFP), Serra Pimenteira, Cachoeira do Cervo, ca. 18°55'S, 54°53'W, ca. 400 m, 22 Feb 1994, *Rodrigues & Pietrobom 733* (PMA, UFP).

##### Discussion.

Alston et al. (1981) considered *Selaginella cabrerensis* endemic to Colombia and Smith (1995), Cremers et al. (2007), and Mostacero (2008) recorded it in Venezuela. However, as already pointed out in Smith (1995), I consider specimens determined as such from Venezuela as a different taxon (i.e., *Selaginella boomii*).

Kessler et al. (2006) considered *Selaginella cabrerensis* as a synonym of *Selaginella xiphophylla* Baker and then described *Selaginella arroyoana* M. Kessler & A.R. Sm. and *Selaginella chiquitana* M. Kessler, A.R. Sm. & M. Lehnert from Bolivia. Later, Huaylla et al. (2010) synonymized *Selaginella chiquitana* under *Selaginella arroyoana*. Valdespino (2015c) suggested that *Selaginella arroyoana* and *Selaginella chiquitana* might be the same taxon as *Selaginella cabrerensis*, and not conspecific with *Selaginella xiphophylla*. After examining type material of all these taxa and SEM studies, I conclude that: a) *Selaginella arroyoana* and *Selaginella chiquitana* are conspecific with *Selaginella cabrerensis*, which has nomenclatural priority; b) *Selaginella cabrerensis* and *Selaginella xiphophylla* are related but distinct taxa as advanced by Valdespino (1993, 2015c); and c) *Selaginella cabrerensis* and *Selaginella xiphophylla* and, at least, *Selaginella densifolia* Spruce, *Selaginella falcata*, and *Selaginella kochii* Hieron., form a morphologically similar alliance (Valdespino 1993) here termed the “*Selaginella falcata* group”. This group is currently under revision (Valdespino in prep.), and a preliminary key to distinguish them is provided below.

I conclude that *Selaginella cabrerensis* is more widely distributed in South America than previously thought, occurring in French Guiana and of more widespread occurrence in Brazil (states of Goiás and Mato Grosso); it was previously known in Brazil only from Mato Grosso do Sul, where it was identified as *Selaginella chiquitana* by Assis and Labiak (2009).

#### Preliminary key to species of the *Selaginella falcata* group

**Table:** 

1	Median leaf apices acute to short-acuminate, if the latter each acumen less than ¼ the length of the lamina.	
2	Lateral leaves strongly imbricate along main stem, the apices obtuse to truncate or broadly acute; median leaves margins hyaline, in a band 4–6 cells wide, the outer bases prominent with 1 or 2 cilia	***Selaginella densifolia***
2'	Lateral leaves distant along main stem, the apices acute; median leaves margins hyaline, in a band 3 cells wide, the outer bases prominent and tufted with 2–5 cilia	***Selaginella cabrerensis***
1'	Median leaf apices long-acuminate to aristate, each acumen or arista ¼–½ the length of the lamina.	
3	Plants ascending to erect; lateral leaves dark brown to atropurpureus with oblique bases; rhizophores axillary to ventro-axillary and restricted to lower half of the stems	***Selaginella xiphophylla***
3'	Plants creeping; lateral leaves golden-brown to green with subcordate to cordate bases; rhizophores ventro-axillary, borne throughout the stems.	
4	Median leaf base subcordate; lateral leaves ovate-oblong, often strongly clasping the stems	***Selaginella kochii***
4'	Median leaf base cordate; lateral leaves oblong-falcate, spreading and free from the stems	***Selaginella falcata***

#### Selaginella chromatophylla

Taxon classificationPlantaeSelaginellalesSelaginellaceae

Silveira

Selaginella chromatophylla Silveira, Bol. Commiss. Geogr. Geol. Minas Geraes 5: 124. 1898. — Type. Brazil. Minas Gerais: Serra do Papagaio, Nov 1897, *A. Silveira s.n., In Herb. Com. Geog. et Geol. Civitatis Minas Geraes No. 2604* (probable holotype: R! [as *Herb. Silveira No.152*, RB 179574]; isotypes: B! [B 20 0129405 (a)], B! [B 20 0129405 (b)]).
Selaginella chromatophylla var. megasperma Silveira, Bol. Commiss. Geogr. Geol. Minas Geraes 5: 125. 1898. — Type: Brazil. Minas Gerais: between Tiradentes and Casa da Pedra, Jun 1898, *A. Silveira s.n., In Herb. Com. Geog. et Geol. Civitatis Minas Geraes No. 2756* (probable holotype: R! [as *Herb. Silveira No.160*, RB 179581]: isotypes: B! [B 20 0129406 (a)], [B 20 0129406 (b)]).
Selaginella breuensis Silveira, Fl. Serras Mineiras 79. 1908. — Type: Brazil. Minas Gerais: Serra do Cipó, Morro do Bréu, Apr 1905, *A. Silveira s.n.* (probable holotype: R! [as *Herb. Silveira No. 395*, RB 179580]: isotype: P [P00573791]-image! [as *Herb. Silveira No. 395*]).

##### Additional specimens examined.

**BRAZIL**. **Bahia**: Palmeiras, Serra dos Brejões, 21 Aug 2009, *Moraes & van der Werff 2861* (MO, PMA, UC). **Minas Gerais**: Cadeia do Espinhaço, Gouveia, near Gouveia-Curvelo, 15 km past Gouveia, Fazenda do Tigre, Córrego da Onça, 18°33'52.3"S, 43°48'22.7"W, 960 m, 17 Mar 2007, *Salino et al.* (BHCB-n.v., PMA); Mpio. Conselheiro Mata, Serra do Espinhaço, between Conselheiro Mata and Diamantina, off MG-220 near km 177, 18.28°S, 43.59°’W, 1290 m, 11 Jan 2010, *Prado et al. 2091* (MO, NY, SP-n.v.), off MG-220 near km 187, -18.27451 [18°16'28.23”]S, -43.71189 [43°42'42.80”]W, 1425 m, 11 Jan 2010, *Schuettpelz et al. 1378* (MO); São Gonçalo do Rio Preto, Parque Estadual do Rio Preto, Córrego das Équas, 18°08'43"S, 43°22'10"W, 8 Apr 200, *Salino et al 5214* (BHCB-n.v., NY); Mpio. Serro, Serra do Espinhaço, between Serro and Milho Verde, off of Estrada Real, ca. 10 km SE of Milho Verde and ca. 10 km NW of Serro, 18°53'S, 43°43'W, 1010 m, 14 Jan 2010, *Prado et al. 2100* (MO, NY, SP-n.v.). **Rio de Janeiro**: Frade de Macahé, 17–21 Jun 1937, *Brade 15824* (MO, RB).

##### Discussion.

*Selaginella chromatophylla* was subsumed under *Selaginella marginata* (Humb. & Bonpl. ex Willd.) Spring by Alston (1936), Reed (1965–1966), Alston et al. (1981), and Hirai and Prado (2000). As currently circumscribed, however, *Selaginella marginata* is morphologically variable and ill-defined. My preliminary revisionary work on *Selaginella marginata*, including SEM studies of leaves and spores, indicates it is a species complex (here termed the “*Selaginella marginata* complex”) that could include, at least, five taxa (Valdespino in prep.). Among these, *Selaginella chromatophylla* and *Selaginella parviarticulata* Buck are very similar because of their small leaf size. *Selaginella chromatophylla* differs from the latter by its lateral and axillary leaves with peltate (vs. basally attached) bases, lateral leaves with acroscopic margins short-ciliate along proximal ½ (vs. entire), median leaves with the peltate auricles ciliate (vs. entire or almost so), and microspores gemmate or globular (vs. corrugate). It differs from typical *Selaginella marginata* by its lateral leaves broadly ovate-elliptic (vs. ovate-deltate) with the peltate auricles ⅙ (vs. ⅓–½) the length of the laminae, both halves of the laminae of equal width (vs. acroscopic halves wider than the basiscopic ones) with obtuse to broadly acute (vs. acuminate) apices, axillary leaves obovate-oblong (vs. ovate-deltate) with obtuse to broadly acute (vs. acuminate) apices, median leaves with acute (vs. long-acuminate) apices, and gemmate or globular (vs. echinulate) microspores. *Selaginella chromatophylla* differs further from the previously discussed *Selaginella marginata* and *Selaginella parviarticulata* by its stems usually branching in a zig-zag pattern.

The name *Selaginella chromatophylla* is provisionally used here for the taxon documented with specimens cited pending a full nomenclatural and taxonomic revision of the rest of the species in the “*Selaginella marginata* complex”. Other names that could potentially apply to that taxon are *Selaginella excurrens* Spring and *Selaginella distorta* (Spring) Spring, which if proven to be conspecific would both have priority over *Selaginella chromatophylla*.

#### Selaginella deltoides

Taxon classificationPlantaeSelaginellalesSelaginellaceae

A. Braun

Selaginella deltoides A. Braun, Ann. Sci. Nat. Bot. ser. 5, 3: 287. 1865. — Syntypes. Brazil. [Amazonas:] prope Panuré, ad Rio Uaupès, *R. Spruce 2532* (B! [B 20 0130962], B! [B 20 0095165 (b)], BM! [BM000905710], BR! [BR00000696525], CGE-n.v., GH! [GH00057068], K! [K000589188], NY-fragment! [NY00022868], OXF!, P! [P00559331], P [P00573927]-image!, RB! [RB 168990], W!); *R. Spruce 2535* (BM!, K!, P [P01244267]-image!, P! [P04026284]).
Selaginella trifurcata Baker, J. Bot. 21: 98. 1883. — Type: Brazil. [Amazonas:] Panuré, on the Rio Uaupès, *R. Spruce 2532* (holotype: K! [K000589188]; isotypes: B! [B 20 0095165 (b)], BM! [BM000905710], BR! [BR00000696525], CGE-n.v., GH! [GH00057067], NY-fragment! [NY00022868], OXF!, P! [P00559331], P! [P00573927]-image!, RB! [RB 168990], W!).

##### Additional specimens examined.

**BRAZIL**. **Amazonas**: Jutica, 14 Nov 1928, *Luetzelburg 23710* (M, R), [Uaupés?], 16 Nov 1928, *Luetzelburg 23731* (M, R), 14 Nov 1928, *Luetzelburg 23735* (R).

##### Discussion.

*Selaginella deltoides* is a much-confused species. Alston (1936) synonymized it under *Selaginella dendricola* Jenman. Reed (1965–1966) treated *Selaginella deltoides* as a valid species and, apparently, subsumed *Selaginella trifurcata* under it. Later, Alston et al. (1981) synonymized *Selaginella deltoides* and *Selaginella trifurcata* under *Selaginella muscosa*. Valdespino (1995) considered *Selaginella deltoides* and *Selaginella trifurcata* as conspecific but not the same species as, or closely related to, *Selaginella muscosa*. In Alston et al. (1981: 298) a possible inadvertent lectotypification for *Selaginella deltoides* and *Selaginella trifurcata*, in the sense outlined by Prado et al. (2015), could be inferred. Furthermore, specimens of *Spruce 2535* at BR! and RB! [RB 168977] are mixed collections which I have determined as: a = *Selaginella dendricola* and b = *Selaginella calceolata* Jermy & Rankin, while the same collection at G! and P! [P04026284] is *Selaginella dendricola*. Another duplicate of *Spruce 2535* at P ([P01244267]-image!) was also determined by Cremers as *Selaginella dendricola*. These nomenclatural and taxonomic matters will be further discussed in a separate paper (see comment about this under *Selaginella glazioviana*).

*Selaginella deltoides* is characterized by its ovate-deltate lateral leaves, with midribs conspicuously hyaline and upper surfaces hispidulous with prickle- or tooth-like projections usually found submarginally, marginally, and apically along the basiscopic halves of the laminae, and median leaves orbiculate or broadly ovate-elliptic. *Selaginella deltoides*, along with a species currently being described with the epithet of “*aculeatifolia*” (Venezuela), *Selaginella brevifolia* (Colombia, Venezuela, and Brazil), and *Selaginella sandwithii* Alston (Guyana and French Guiana) form a closely related group of taxa referred as the “*Selaginella deltoides* group” (Valdespino in press).

#### Selaginella falcata

Taxon classificationPlantaeSelaginellalesSelaginellaceae

(P. Beauv.) Spring

Selaginella falcata (P. Beauv.) Spring, Bull. Acad. Roy. Sci. Bruxelles 10: 225. 1843. — *Stachygynandrum falcatum* P. Beauv. Mag. Encycl. 9^e^ Année, 5: 483. 1804. — Type. French Guiana. [without date or precise locality], [*A.*?] *Chastelein s.n.* (OXF-n.v.).

##### Additional specimens examined.

**BRAZIL**. **Amapá**: Rio Pontanari, 03°45'N, 51°42'W, 31 Jul 1960, *Irwin et al. 47271* (NY, UC); 1956, *Bastos 70 [2070]* (RB), *Bastos 2070* (RB).

##### Discussion.

In Alston et al (1981), *Selaginella falcata* was documented only in French Guiana; here its range is extended to include Brazil. This is a very distinct species because of its creeping to prostrate habit, ovate-deltate or deltate median leaves, large oblong, lateral leaves each 4–7.5 mm long, and main stem width (including lateral leaves) 8.0–15 mm. In Brazil, *Selaginella falcata* may be confused with *Selaginella mendocae* Hieron., because of their similar habit and stem width (including lateral leaves). However, the former differs from the latter by ovate-deltate or deltate (vs. orbiculate) median leaves, with both halves equal in width (vs. outer halves wider than inner ones), and leaf bases cordate (vs. bases oblique or inner ones truncate and outer bases lobed). Another species with similar median leaves as those of *Selaginella falcata* is *Selaginella kochii*. These two taxa are part of the *Selaginella falcata* group and can be separated by the characters provided in the key under *Selaginella cabrerensis*.

#### Selaginella glazioviana

Taxon classificationPlantaeSelaginellalesSelaginellaceae

Hieron

Selaginella glazioviana Hieron., Hedwigia 43: 36. 1904. — Type. Brazil. [Rio de Janeiro: Nova Friburgo, Alto Macahé, chez les Crannin, 21 Jan 1874], *A. Glaziou 7280* (holotype: B! [B 20 0095195]; isotypes: BM-fragment! [BM000905698], P [P00559320]-image!, P [P00559321]-image!).

##### Additional specimens examined.

**BRAZIL**. **Minas Gerais**: Serra do Espinhaço, Pico de Itacolomi, 3 km S of Ouro Preto, 1600 m, 1 Feb 1971, *Irwin et al. 29530* (F, K, NY), *29554* (K, NY); Ouro Preto, Chapada, 20°28'53.8"S, 43°33'03.6"W, 1100 m, 6 Nov 2006, *Salino & Salino 14999* (BHCB-n.v., PMA); Ouro Preto, São Bartolomeu, 20°15'34.44"S, 43°34'50.56"W, 1035 m, 13 Oct 2007, *Dittrich & Lobão 1499* (BHCB-n.v., PMA); Santa Bárbara, RPPN Capivari, along path to Cachoeira de Capivari, 20°07'57"S, 43°34'54"W, 1140 m, 12 Dec 2009, *Arruda & Filogonio 23* (BHCB-n.v., PMA), West portion of the RPPN, entrance to Fazenda do Zé Maria, 20°07'45"S, 43°35'50"W, 900 m, 1 May 2009, *Arruda et al. 127* (BHCB-n.v., PMA); Serra do Itacolomy, 1936, *Badini 316* (BM).

##### Discussion.

Alston (1936), Reed (1965–1966), and Alston et al. (1981) included *Selaginella glazioviana* under *Selaginella erectifolia*. I have studied type material of *Selaginella erectifolia* (*Swainson s.n.*, holotype: K!) and its synonym *Selaginella camptostachys* Fée (*Glaziou 2242*, holotype: P!; isotypes: B!, BR!, K!, NY-fragment!) and agree with Alston et al. (1981) that they are conspecific, but I consider *Selaginella glazioviana* to be a different species. A full account of that finding, along with the formal resurrection of *Selaginella deltoides* and *Selaginella chromatophylla*, will be reported separately (Valdespino in prep). *Selaginella glazioviana* is morphologically close to *Selaginella trygonoides*, described herein (see for comparison).

#### Selaginella lechleri

Taxon classificationPlantaeSelaginellalesSelaginellaceae

Hieron

Selaginella lechleri Hieron., in Engler & Prantl, Nat. Pflanzenfam. 1 (4): 683. 1901. — Type. Peru. Puno: Near San Gaván, Jul 1854, *W. Lechler 2159* (lectotype: B! [B 20 0095276], designated by R. Tryon and Stolze (Fieldiana Bot. n.s. 34: 79. 1994); isolectotypes: BM-fragment! [BM000905693], K-fragment! [K001044504], P! [P00044863]), P [P00573771]-image!, P-fragment [P00573772]-image!.

##### Additional specimens examined.

**BRAZIL**. **Acre**: Cruzeiro do Sul, Serra do Divisor, 07°26'53"S, 73°40'00"W, 13–14 Dic 2007, *Brasil et al. 315* (RB), Mâncio Lima, Parque Nacional da Serra do Divisor, Bacia do Alto Juruá, Río Moa, Parque Nacional da Serra do Divisor, 07°26'S, 73°39'41"W, 16 Jun 1996, *Silveira et al. 1364* (NY), Serra do Divisor, 07°27'13.4"S, 73°41'30"W, 240 m, 23 Aug 2008, *Fiaschi et al. 3387* (NY, RB), Serra deo Moa, 07°28'00"S, 73°37'27"W, 6 May 1996, *Daly et al. 8879* (NY), Faz. Arizona (30 min de canoa a motor rio abaixo da Serra do Moa), ca. 07°30'S, 73°40'W, 5–7 Oct 1985, *Jangoux et al. 85-104* (MG, NY). **Amazonas**: Benjamim Constant, Alto Solimões, 9 Sep 1962, *Duarte 6586* (RB); Rio Javari, 8 mi above mouth of Rio Curaçá, 26 Oct 1976, *Prance et al. 24131* (K, NY); Río Juruá, Fortaleza, Oct 1901, *Ule 6931* (L); Río Negro, between Manaus and São Gabriel, along BR 307, N of Igarapé Iá-Mirim, near Jerusalém, 00°20'N, 66°35'W, 17 Jul 1979, *Poole 2037* (MG, NY).

##### Discussion.

*Selaginella lechleri* is known from Colombia and Peru (Alston et al. 1981). Additionally, Smith (1995) cited it in Costa Rica, Panama, Venezuela, and Bolivia, while Cremers et al. (2007) and Mostacero (2008) also cited it in Venezuela, and Tropicos (2015a) registered it from Ecuador. I believe that specimens from Costa Rica and Panama are most likely *Selaginella anceps* (C. Presl) C. Presl, while those from Venezuela are best referred to another species. *Selaginella lechleri* is documented here to occur in the states of Acre and Amazonas, Brazil. It is characterized by its erect habit, lateral leaves with the basiscopic bases geniculate and usually glabrous, with the acroscopic margins short-ciliate along proximal ⅓ or dentate, otherwise sparsely denticulate to entire or serrate distally, and acuminate median leaves.

#### Selaginella microdonta

Taxon classificationPlantaeSelaginellalesSelaginellaceae

A.C. Sm.

Selaginella microdonta A.C. Sm., Bull. Torrey Bot. Club 58: 313. 1931. — Type. Venezuela. Amazonas: on slope of Ridge 24 and summit of Mount Duida, 1800 m, Aug 1928–Apr 1929, *G.H.H. Tate 509* (holotype: NY! [NY00144140]; isotypes: BM [BM000936552]-image!, US-fragment! [US00135732]).

##### Additional specimen examined.

**BRAZIL**. **Amazonas**: Alto Río Negro, Cucui, Serra Tunui, 29 Apr 1975, *Cavalcante 3056* (MG).

##### Discussion.

*Selaginella microdonta* was previously known only from Venezuela (Alston et al. 1981, Smith 1995, Cremers et al. 2007, Mostacero 2008); its range is now extended to include Brazil. This species is characterized by being a minute, creeping plant with coriaceous leaves, the median leaves ovate, ovate-deltate with oblique to rounded bases with the outer ones prominent. Furthermore, these leaves have prominent, often arcuate midribs (making the halves of the laminae unequal), serrate to serrulate margins, and acute apices; the lateral leaves are broadly ovate-elliptic with thickened margins, these denticulate to entire, and acute apices tipped by 1–3 teeth. In Brazil, *Selaginella microdonta* may be confused with *Selaginella wurdackii*, from which it differs by its creeping habit and smaller median leaves with acute apices. According to Valdespino (1992), *Selaginella microdonta* is morphologically related to *Selaginella cardiophylla* Valdespino and *Selaginella hemicardia* Valdespino. The possible interrelationships and distinguishing characters are further discussed in Valdespino (1992).

#### Selaginella potaroensis

Taxon classificationPlantaeSelaginellalesSelaginellaceae

Jenman

Selaginella potaroensis Jenman, Gard. Chron., ser. 3, 2: 154. 1887. — Type. Guyana. Essequibo: [ravines near the foot of the] Kaieteur Falls, [04°47’ 31.26"N, 58°58'29.21"W], [Sep 1881], *G.S. Jenman 1818* (lectotype: NY! [NY00144158], designated by Alston et al. (Bull. Brit. Mus. (Nat. Hist.), Bot. 9: 297. 1981); isolectotypes: BM [BM000634459]-image!, K! [K000589087]).

##### Additional specimens examined.

**COSTA RICA**. **Heredia**: between Río Peje and Río Sardinalito, Atlantic slope of Volcán Barba, 10°17.5'N, 84°04.5'W, 700–750 m, 4 Apr 1986, *Grayum 6743* (MO); Parque Nacional Braulio Carrillo, transect between Volcán Barba and OET station La Selva, 600 m, 2003, *Kluge 2234* (PMA). **BRAZIL**. **Roraima**: Serra dos Surucucus, NE of Mission station, 02°42–47'N, 63°33–36'W, 1000–1400 m, 17 Feb 1969, *Prance et al. 9995 (NY)*.

##### Discussion.

*Selaginella potaroensis* was previously known to occur in Venezuela, Guyana, Suriname, and French Guiana (Alston et al. 1981, Smith 1995, Cremers et al. 2007). Its range is documented here northward to include Costa Rica (see Tropicos 2015b), and it is to be expected in Panama and Colombia. This species is also known from Brazil.

*Selaginella potaroensis* is a creeping plant characterized by having axillary rhizophores throughout the stems, membranaceous leaves, the median leaves semicordate to ovate with the outer half of the lamina almost twice as wide as the inner one, margins hyaline and denticulate with the outer margin convex and the inner one almost straight, laminae abruptly tapering into a long-acuminate apex with conspicuous stomata on the upper surface, the lateral leaves ovate-elliptic with minute submarginal teeth along the upper surfaces of basiscopic halves, and acute apices tipped by 1–3 teeth. The similarities and differences of *Selaginella potaroensis* with *Selaginella cardiophylla*, *Selaginella hemicardia*, and *Selaginella rhodostachya* Baker are discussed in Valdespino (1992).

#### Selaginella seemannii

Taxon classificationPlantaeSelaginellalesSelaginellaceae

Baker

Selaginella seemannii Baker, J. Bot. 21: 244. 1883. — Type. Nueva Granada [Colombia]. Chocó: Cacagual [Island], 1848, *B.C. Seemann 1006* (holotype: K! [K000589106]; isotypes: B-fragment! [B 20 0095503], BM! [BM000936546]).
Selaginella barbacoasensis Hieron., Hedwigia 43: 46. 1904. — Type: Colombia. Nariño: Barbacoas, [03°54'04.16"N, 73°04'24.13"W], 6 Aug 1880, *F.C. Lehmann 89* (holotype: B! [B 20 0130329]; isotypes: BM! [BM000936545], G!).

##### Additional specimens examined.

**PANAMA**. **Coclé**: Bosque cercano a Coclecito, 28 Mar 1989, *Galdames et al. 362* (PMA); Cerro Morenito cerca de Coclecito, 24.8 km desde la entrada de Llano Grande hacia Coclecito, 230 m, 10 Jan 1992, *Valdespino et al. 1357* (BM, CR, K, MO, NY, PMA, UC, US). **Veraguas**: Santa Fe, Parque Nacional Santa Fe, El “2”, entrando por Guinea (Río Concepción), UTM Zone 17: 0963119N [8°42'46.68"N], 0501054E [80°59'25.50"W], 22 Jul 2008, *Hernández 989* (PMA). **BRAZIL**. **Roraima**: Boa Vista, Reserva Ecológica de Maracá, track to Santa Rosa, ca. 600 m E of main Estação buildings, ca. 80 m, 12 Mar 1987, *Edwards & Willikin 2541* (BM, NY).

##### Discussion.

*Selaginella seemannii* is known to occur in Colombia, Guyana, Suriname, French Guiana, Ecuador, and Peru (Alston et al. 1981, Cremers et al. 2007). It is also found in Panama and Brazil (Valdespino 1995) and here is formally vouchered for those countries.

*Selaginella seemannii* is similar to and may be confused with *Selaginella porelloides* (Lam.) Spring. *Selaginella seemannii* can be distinguished by its ovate (vs. semicordate) lateral leaves with the upper surfaces glabrous throughout (vs. pubescent near the basiscopic margins), obtuse (vs. narrowly obtuse to slightly acute) apices, lower leaf surfaces with obscure (vs. conspicuous) idioblasts, bases of median leaves oblique with an outer auricle (vs. cordate to rounded, non-auriculate), and bases of axillary leaves rounded (vs. usually cordate).

#### Selaginella umbrosa

Taxon classificationPlantaeSelaginellalesSelaginellaceae

Lem. ex Hieron.

Selaginella umbrosa Lem. ex Hieron., in Engler & Prantl, Nat. Planzenfam. 1(4): 683. 1901. — Type. Guatemala. [exact locality and date unknown], *G.U. Skinner s.n* (K-n.v.).

##### Additional specimens examined.

**BRAZIL**. **Roraima**: vicinity of Auaris, 04°03'N, 64°22'W, 760–800 m, 6 Feb 1969, *Prance et al. 9653* (NY, MG, P-image); Rio Catrimani, Ponto 12, 70 km N of Missão Catrimani, 13 Feb 1975, *Pires 15* (MG, RB); Rio Uraricoera, Canal Maracá, Cachoeira Menori, 03°16'N, 61°55'W, 24 Feb 1979, *Pires et al. 16789* (MG); SEMA Ecological Reserve, Ilha de Maracá, river near the Casa da Maracá, 11 May 1987, *Milliken 195* (BM-n.v., INPA, MIRR-n.v.). **Without precise locality**: Mountain from North of Brasil, s.d., s.col. *ex Herb. Gaillary* (P-image).

##### Discussion.

*Selaginella umbrosa* occurs in Central America, Barbados, Colombia, Trinidad, and Tobago (Alston et al. 1981, Fraile 1995, Smith 1995, Cremers et al. 2007, Mostacero 2008), and Ecuador (Tropicos 2015a). It is here documented from Brazil, were it may be confused with *Selaginella haematodes* (Kunze) Spring because of its red stems and leaves. As pointed out by Valdespino (1993), *Selaginella umbrosa* differs from the latter by its lateral leaves with acroscopic margins ciliate, especially on proximal ½ (vs. obscurely serrulate to entire), and median leaves broadly ovate to ovate-lanceolate (vs. lanceolate to lanceolate-oblong), the bases with outer, ciliate auricles (vs. bases rounded to oblique without outer auricles).

#### Selaginella vernicosa

Taxon classificationPlantaeSelaginellalesSelaginellaceae

Baker

Selaginella vernicosa Baker, Timehri 5: 220. 1886. — Type. Venezuela. Bolivar: SE slopes of Mount Roraima, “Our House”, 1622 m, *E.F. im Thurn 226* (holotype: K! [K000589193]; isotype: US! [US00135747]).
Selaginella vernicosa var oligoclada Baker, Trans. Linn. Soc. Lond. Bot. 2: 295. 1887. — Type: Venezuela. Bolivar: SE slopes of Mount Roraima, “Our House”, 1622 m, Dec 1884, *E.F. im Thurn 381* (holotype: K! [K000589194]; isotypes: BM! [BM001099755], US! [US 00135748]).

##### Additional specimens examined.

**BRAZIL**. **Roraima**: 2300 m, Dec 1909, *Ule* 8492 (K, MO), 2850 m, Oct 1927, *Luetzelburg 21631* (R), *21639* (R).

##### Discussion.

For a long time, *Selaginella vernicosa* was thought to occur only in Venezuela (Alston et al. 1981, Smith 1995), but Cremers et al. (2007) reported it recently from Guyana, a report that needs to be confirmed. Its range is also here documented in Brazil.

*Selaginella vernicosa* is most distinct by its ascending to erect habit, coriaceous, strongly imbricate and shiny leaves due to waxy deposits, the median leaves ovate to ovate-deltate with the midribs raised or prominent, with short-ciliate margins at least along proximal ⅔, otherwise dentate on distal ⅓, carinate, acute apices, lateral leaves ovate-deltate with acroscopic margins short-ciliate along proximal ½, otherwise dentate distally, and basiscopic margins ciliate along proximal ¼–½, otherwise entire distally with the apices entire or tipped by a tooth.

#### Selaginella wurdackii

Taxon classificationPlantaeSelaginellalesSelaginellaceae

Alston

Selaginella wurdackii Alston, Bull. Brit. Mus. (Nat. Hist.), Bot. 9: 280. 1981. — Type. Venezuela. Bolivar: Chimantá Massif, Río Tirica, La Laja Base Camp, 485–490 m, *J.A. Steyermark & J.J. Wurdack 173* (holotype: BM [BM000936544]-image!; isotypes: F!, NY! [NY00144232 (a)], US! [US00433011]).

##### Additional specimens examined.

**VENEZUELA**. **Bolivar**: near Salto de Pacairo, NE of Santa Teresita de Kavanayén, 1220 m, *Steyermark 60499* (BM-n.v., F). **BRAZIL**. **Amazonas**: São Gabriel da Cachoeira, Parque Nacional do Pico da Neblina, trail to Pico Neblina, between Camp Bebedouro Velho and Bebedouro Novo, 00°44'13"N, 65°57'14"W, 26 Dec 2004, *Carvalho et al. 233* (INPA-n.v., PMA).

##### Discussion.

*Selaginella wurdackii* was previously known only from Venezuela (Alston et al. 1981, Smith 1995, Cremers et al. 2007, Mostacero 2008) and is reported here for the first time from Brazil. It is characterized by its ascending to erect habit with axillary rhizophores, median leaves ovate, narrowly ovate-deltate to ovate-lanceolate with prominent or keeled midribs that may be displaced (i.e., curved) to one side making the halves of the laminae unequal, leaf margins greenish to faintly hyaline, seemingly entire or denticulate along distal ½ and with long-acuminate to short-aristate apices, and lateral leaves ovate-elliptic with margins thickened and apices acute to short-acuminate.

## Supplementary Material

XML Treatment for Selaginella nanuzae

XML Treatment for Selaginella neospringiana

XML Treatment for Selaginella pellucidopunctata

XML Treatment for Selaginella stomatoloma

XML Treatment for Selaginella trygonoides

XML Treatment for Selaginella beitelii

XML Treatment for Selaginella cabrerensis

XML Treatment for Selaginella chromatophylla

XML Treatment for Selaginella deltoides

XML Treatment for Selaginella falcata

XML Treatment for Selaginella glazioviana

XML Treatment for Selaginella lechleri

XML Treatment for Selaginella microdonta

XML Treatment for Selaginella potaroensis

XML Treatment for Selaginella seemannii

XML Treatment for Selaginella umbrosa

XML Treatment for Selaginella vernicosa

XML Treatment for Selaginella wurdackii
